# Meta-analysis of the prognostic value of circulating tumor cells detected with the CellSearch System in colorectal cancer

**DOI:** 10.1186/s12885-015-1218-9

**Published:** 2015-03-30

**Authors:** Xuanzhang Huang, Peng Gao, Yongxi Song, Jingxu Sun, Xiaowan Chen, Junhua Zhao, Huimian Xu, Zhenning Wang

**Affiliations:** Department of Surgical Oncology and General Surgery, First Hospital of China Medical University, 155 North Nanjing Street, Heping District, 110001 Shenyang City, People’s Republic of China

**Keywords:** Circulating tumor cells, Colorectal cancer, CellSearch System, Prognosis, Meta-analysis

## Abstract

**Background:**

The prognostic value of circulating tumor cells (CTCs) detected with the CellSearch System in patients with colorectal cancer (CRC) is controversial. The aim of our meta-analysis was to evaluate whether the detection of CTCs in the peripheral blood with the standardized CellSearch System has prognostic utility for patients with CRC.

**Methods:**

The PubMed, Science Citation Index, Cochrane Database, Embase, and the references in relevant studies were systematically searched (up to December, 2014). No search restrictions were imposed. Our meta-analysis was performed in Stata software, version 12.0 (2011) (Stata Corp, College Station, TX, USA), with the odds ratio (OR), risk ratio (RR), hazard ratio (HR), and 95% confidence interval (95% CI) as the effect measures. Subgroup and sensitivity analyses were also conducted.

**Results:**

Eleven studies containing 1847 patients with CRC were analyzed. There was a significantly higher incidence of CTCs in the metastasis-positive group than in the metastasis-negative group (OR = 4.06, 95% CI [1.74, 9.50], P < 0.01, I^2^ = 0%). For hepatic metastasis, a type of metastasis, a higher incidence of CTCs was observed in the hepatic-metastasis-positive group than in the -negative group (OR = 2.61, 95% CI [1.73, 3.96], P < 0.01, I^2^ = 0%). The presence of CTCs was significantly related to overall survival (HR = 2.00, 95% CI [1.49, 2.69], P < 0.01, I^2^ = 67.1%) and progression-free survival (HR = 1.80, 95% CI [1.52, 2.13], P < 0.01, I^2^ = 43.9%) of patients with CRC, regardless of the sampling time. The response rate for the CTC^+^ groups was significantly lower than that for the CTC^−^ groups at baseline and during treatment (baseline: 33% versus 39%, RR = 0.79, 95% CI [0.63, 0.99], P = 0.04, I^2^ = 7.0%; during treatment: 17% versus 46%, RR = 0.41, 95% CI [0.22, 0.77], P = 0.01, I^2^ = 0.0%;).

**Conclusions:**

Our meta-analysis indicates that the detection of CTCs in the peripheral blood with the CellSearch System has prognostic utility for patients with CRC.

**Electronic supplementary material:**

The online version of this article (doi:10.1186/s12885-015-1218-9) contains supplementary material, which is available to authorized users.

## Background

Colorectal cancer (CRC) is the third most commonly diagnosed cancer in males and the second in females worldwide, with over 1.2 million new cases and 608,700 deaths estimated to have occurred [1]. Recurrence and metastasis are still the main reasons for CRC-related deaths, although awareness of CRC has increased and its treatment improved in recent years [2]. The liver is the most frequent metastatic site, and metastasis to the liver can occur through the portal system [3]. However, the detailed mechanisms of the metastatic cascade of CRC are yet to be clarified. Today, it is accepted that circulating tumor cells (CTCs), which are released into the blood circulation from the primary tumor, play an important role in the formation of metastases, according to the “seed and soil theory” [4].

Several studies have shown that the presence of CTCs in the blood circulation is a poor prognostic indicator of overall survival (OS) and progression-free survival (PFS) in patients with CRC [5,6]. In those studies, the diagnostic methods used to detect CTCs were predominantly reverse transcription–polymerase chain reaction (RT–PCR) [7] and immunocytochemistry (ICC) [8], targeting either tumor-associated genes or antigens. However, the detection methods vary across laboratories and the optimal cut-off value for CTCs has not yet been confirmed. Currently, the CellSearch System (Veridex, Raritan, NJ, USA), a semiautomated immunomagnetic method for the quantification of CTCs based on the epithelial cell adhesion molecule (EpCAM), is the first standardized system approved by the U.S. Food and Drug Administration [9] and has been used to detect CTCs in patients with breast, prostate, and colorectal cancer [10-12]. From a clinical perspective, using CTCs detected in the peripheral blood (PB) to evaluate the prognosis of cancer is the least invasive procedure for patients, and is more time effective and repeatable than assays of the bone marrow or mesenteric/portal blood. However, for clinical applications, the prognostic utility of CTCs detected with CellSearch in CRC patients has not yet been consistently determined [12-15]. Therefore, a pooled analysis of available studies that have used the CellSearch System is required to assess the prognostic relevance of CTC detection in the PB of patients with CRC.

The aim of our study was to use a meta-analysis to quantitatively and comprehensively summarize the prognostic significance of CTCs detected with the standardized CellSearch System in patients with CRC.

## Methods

### Search strategy

A literature search for relevant studies was performed systematically (up to December 2014). The following databases were searched: PubMed, Science Citation Index, Cochrane Database, and Embase databases. The reference lists of the relevant studies (review studies and included studies) were also checked for potentially relevant articles. The main keywords and MeSH terms used were: “circulating tumor cells”, “micrometastasis”, “disseminated tumor cells”, “isolated tumor cells”, “occult tumor cells”, “peripheral blood”, “colorectal cancer”, “colon cancer”, “rectal cancer”, “gastrointestinal cancer”, and “CellSearch System” (Additional file 1).

### Inclusion criteria

To be included in our meta-analysis, eligible studies had to fulfill the following criteria: (1) investigated the clinicopathological or prognostic significance of CTC detection in CRC patients, with at least one of the outcome measures of interest reported in the study or calculable from the published data; (2) used only the CellSearch System to detect CTCs; and (3) collected the samples from the PB. When multiple studies were published by the same patient population, we included the most informative study.

### Exclusion criteria

Studies were excluded from the meta-analysis if: (1) the samples came from the bone marrow, mesenteric/portal blood, lymph nodes, or peritoneal cavity; (2) the number of patients with CRC analyzed in all pooled analyses was less than 20; (3) the outcomes of interest were not reported or could not be calculated from the original published data; and (4) the study was redundant, based on the same database or patients population as an included study. To avoid the inclusion of redundant studies, all the included studies were checked carefully, including their authors, organizations, the accrual period, and the population of patients.

### Data extraction

Two reviewers (XZ Huang and P Gao) reviewed each of the studies included, and extracted the data independently. The following information was collected: first author, year of publication, country, characteristics of the study population (i.e., number, sex, age, accrual period, population), chemoradiotherapy (postoperative or palliative for inoperable patients), sample time, rate of CTC positivity, follow-up period, cut-off point, prognostic outcomes (overall survival [OS] and progression-free survival [PFS]), hazard ratio (HR), and the objective response to adjuvant chemotherapy (but not to neoadjuvant chemotherapy), according to the Response Evaluation Criteria In Solid Tumors (RECIST) guideline (complete response [CR], partial response [PR], stable disease [SD], and progressive disease [PD]) [16]. Disagreements were resolved by discussion between the two reviewers.

### Assessment of the risk of bias

Two reviewers (XZ Huang and P Gao) used the Newcastle–Ottawa Scale (NOS) criterion [17], which is used for nonrandom controlled trials (non-RCTs), to independently evaluate the quality of the included studies. The results of quality assessment were confirmed through the agreement between the two reviewers in the quality assessment. Any disagreements on quality assessment were resolved via comprehensive discussion. The NOS is based on three aspects of the study: selection, comparability, and outcome.

### Statistical methods and subgroup/sensitivity analysis

Our meta-analysis was completed according to the recommendations of the Preferred Reporting Items for Systematic Reviews and Meta-analyses (PRISMA) [18]. The meta-analysis of test accuracy data was conducted by Meta-DiSc (Version 1.4) [19], and the remaining statistical analyses were performed in Stata software, version 12.0 (2011) (Stata Corp, College Station, TX, USA). The estimated odds ratio (OR) was used to summarize the association between the detection of CTCs and the clinicopathological characteristics of CRC. The risk ratio (RR) and hazard ratio (HR) were used to summarize the effect measures for the prognostic outcomes (objective response, PFS, and OS).

If the HR and its variance were not provided directly by an included study, we calculated these values from the available data with the method designed by Jayne F Tierney [20]. By convention, HR, RR, or OR > 1 indicated an unfavorable outcome in the CTC^+^ group compared with the CTC^−^ group. According to the RECIST guideline, we assessed the sensitivity and specificity of CTC^+^ in predicting objective response to chemotherapy (no-response events [SD + PD] and disease progression events [PD]), assuming radiographic imaging to be the gold standard. In the term of sample time, baseline defined as the time before the initiation of any first-line or subsequent systemic chemotherapy, and during-treatment was defined as the time during the course of systemic chemotherapy (including first line or subsequent systemic chemotherapy). All statistical values were reported with 95% confidence intervals (95% CIs) and the two-side P value threshold for statistical significance was set at 0.05. Heterogeneity among the studies was calculated with the Q test and I^2^ statistic [21], and the I^2^ value indicated the degree of heterogeneity. A P value <0.10 for the Q statistic and/or I^2^ > 50% were considered significant heterogeneity, and a random-effects model was used. Otherwise, a fixed-effects model was used. Meta-regression was performed to explore the potential variables that contributed heterogeneity. Besides, Galbraith plot was also used to explore which study would contribute substantial heterogeneity to our meta-analysis.

To retain maximum information, we combined multiple effect values into a pooled estimate for further analysis if one study reported several results separately for different sampling time points. We added additional information into included study from original authors or excluded studies if the included and excluded studies were based on the same patients’ population and some information of interest was reported in the excluded studies but not in the included studies. The overall analysis was completed by enrolling all the relevant studies according to the different prognostic outcomes and clinicopathological parameters. A simultaneous subgroup analysis was performed based on the sampling time (baseline or during-treatment). To assess the stability and consistency of our results and to investigate the impact of single study on results, sensitivity analyses were conducted by using the leave-one-out approach (omitting each study individually). Besides, additional subgroup analysis for cut-off values also was conducted. Publication bias was assessed by Egger’s and Begg’s tests [22,23].

## Results

### Baseline characteristics of the included studies

**Figure 1:**
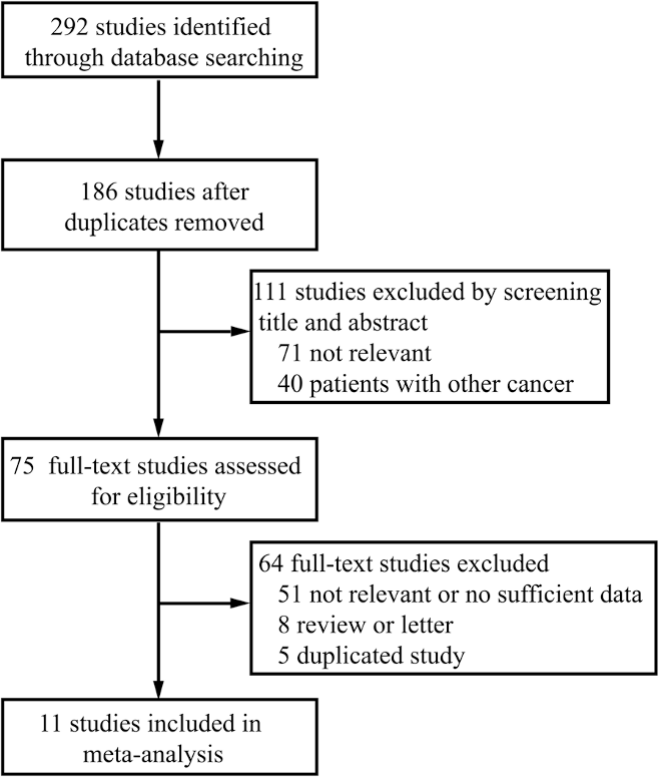
**Selection of studies.** Flow chart showing the selection process for the including studies.

A total of 186 studies were initially identified with the keywords used to search the databases during a systematic literature search. By screening the titles and abstracts, Seventy-five potential studies were retrieved. Sixty-four studies were then excluded after they were fully reviewed because they were review (8 studies), irrelevant or lacked the outcome of interest (51 studies), or redundant (5 studies). Finally, eleven studies met the inclusion criteria and were eligible for our meta-analysis (Figure 1).

The eleven studies included contained 1847 patients with CRC (median sample size: 119 [20–472]; mean: 168) [13-15,24-31]. The studies were conducted in Asia, Europe, and North America (Japan, Germany, Denmark, Spain, France, Netherlands, Norway, Italy and America) and were published between 2008 and 2014. According to the sampling time points, six studies only assessed CTCs at baseline [13,14,25,26,29,30], one study only assessed CTCs at the during-treatment time point [24], and three studies assessed CTCs at baseline and during-treatment time point separately [27,28,31], and one study assessed CTCs combined at both time points [15]. HRs for OS and PFS were provided by nine [14,15,24,25,27-31] and eight [14,24-28,30,31] of the studies, respectively. The baseline characteristics of the included studies and the study design variables are summarized in Table 1. The quality of the eight included cohort studies was evaluated according to NOS and is summarized in Table 2.

**Table 1:** **Baseline characteristics and design variables of the including studies**

Article	Number (M/F)^1^	C/R/R-S^2^	Age Mean ± SD^3^/Median (range)	ST^4^	Cut-off	Rate(+)^5^	Follow up Mean ± SD/Median (range)	OM^6^	Surgery	MA^7^
Sotelo 2014 [24]	472(254/218)	425/47/0	Median:66(31–87)	Baseline	≥1/7.5 ml	166/472	Median:40(NR^8^)	OS^9^; PFS^10^	YES	NO
	472(254/218)	425/47/0	Median:66(31–87)	Baseline	≥2/7.5 ml	93/472	Median:40(NR)	OS; PFS	YES	NO
	472(254/218)	425/47/0	Median:66(31–87)	Baseline	≥3/7.5 ml	57/472	Median:40(NR)	OS; PFS	YES	NO
	472(254/218)	425/47/0	Median:66(31–87)	Baseline	≥5/7.5 ml	34/472	Median:40(NR)	OS; PFS	YES	NO
Seeberg 2014 [25]	194(105/89)	124/70	Median:65(31–93)	Baseline	≥1/7.5 ml	37/189	Median:22.5(1–61)	OS; PFS	153YES	NO
	194(105/89)	124/70	Median:65(31–93)	Baseline	≥2/7.5 ml	26/189	Median:22.5(1–61)	OS; PFS	153YES	YES
	194(105/89)	124/70	Median:65(31–93)	Baseline	≥3/7.5 ml	17/189	Median:22.5(1–61)	OS; PFS	153YES	NO
Gazzaniga 2013 [26]	119(68/51)	NR	Median:64(29–84)	Baseline	≥1/7.5 ml	44/119	Median:12(1–26)	PFS	NR	YES
	119(68/51)	NR	Median:64(29–84)	Baseline	≥3/7.5 ml	24/119	Median:12(1–26)	PFS	NR	YES
Aggarwal 2013 [27]	Baseline:209(NR)	NR	Mean:63.0 ± 12.6 Median: 64 (22–92)	Baseline	≥3/7.5 ml	62/209	median: NR(0.2-39.1)	OS	NR	YES
	3-5 W:115(NR) 6-12 W:134(NR)	NR	NR	During-treatment: 3-5 W,6-12 W^11^	≥3/7.5 ml	3-5 W: 17/115; 6-12 W: 10/134	NR	OS	NR	YES
Kuboki 2013 [14]	63(34/29)	41/22/0	Median: 61(33–81)	Baseline	≥3/7.5 ml	19/63	Median:8.7(NR)	OS; PFS	NR	YES
Deneve 2013 [13]	69(43/26)	66/8/1	Median: 75(38–95)	Baseline	≥1/7.5 ml	20/69	Mean:31 ± NR Median:36 (0–52)	NR	YES	NO
Sastre 2012 [28]	Baseline:180(118/62)	40/121/19	Median: 65(40–82)	Baseline	≥3/7.5 ml	85/180	NR	OS; PFS	123YES	YES
	Cycle3:147(NR)	NR	NR	Cycle3	≥3/7.5 ml	23/147	NR	OS; PFS	123YES	NO
Sato 2012 [29]	25(NR)	NR	NR	Baseline	≥3/7.5 ml	14/25	NR	OS	M1:NO^12^	NO
	25(NR)	NR	NR	Baseline	≥1/7.5 ml	18/25	NR	OS	M1:NO	NO
Papavasiliou 2010 [30]	20(13/7)	NR	Median: 54 (41–81)	Baseline During-treatment	≥3/7.5 ml	Pre:2/20 intra: 10/20; post: 1/18	Median:11.5 (5–25)	OS; PFS	YES	NO
Tol 2010 [31]	467 (284/183)	225/122/ 120	Median: 63(27–83)	Baseline	≥3/7.5 ml	Baseline: 129/451	Median: 16.8(NR)	OS; PFS	NR	YES
	1-2 W: 368(NR) 3-5 W:320(NR) 6-12 W:336(NR) 13-20 W: 254(NR)	NR	Median: 63(27–83)	During-treatment: 1-2/3-5/6-12/13-20 W	≥3/7.5 ml	1-2 W: 21/368; 3-5 W: 17/320; 6-12 W: 18/336; 13-20 W: 16/254	NR	OS; PFS	NR	YES
Hiraiwa 2008 [15]	40(NR)	NR	NR	Baseline + During-treatment	≥2/7.5 ml	14/40	NR	OS	YES	YES

^1^M/F: Male/female.

^2^C/R/R-S: Colon/Rectum/Rectosigmoid.

^3^SD: Standard deviation.

^4^ST: Sampling time.

^5^Rate(+): Rate of CTCs positive patients, n/N.

^6^OM: Outcome measured.

^7^MA: Multivariance analysis.

^8^NR: Not reported.

^9^OS: Overall survival.

^10^PFS: Progression-free survival.

^11^W: Week.

^12^M1: Tumor metastasis positive.

**Table 2:** **The assessment of the risk of bias in each Cohort study using the Newcastle-Ottawa scale**

Study	Selection (0-4)				Comparability(0-2)		Outcome (0-3)			Total
	REC	SNEC	AE	DO	SC	AF	AO	FU	AFU	
Sotelo 2014 [24]	*	*	*	*	-	-	*	*	-	6
Seeberg 2014 [25]	*	*	*	*	-	-	*	*	*	7
Gazzaniga 2013 [26]	-	*	*	*	-	-	*	-	-	4
Aggarwal 2013 [27]	-	*	*	*	-	-	*	*	-	5
Kuboki 2013 [14]	-	*	*	*	-	-	*	*	-	5
Deneve 2013 [13]	-	*	*	*	-	-	*	*	-	5
Sastre 2012 [28]	*	*	*	*	-	-	*	*	-	6
Sato 2012 [29]	*	*	*	*	-	-	*	-	-	5
Papavasiliou 2010 [30]	-	-	*	*	-	-	*	-	-	3
Tol 2010 [31]	*	*	*	*	-	-	*	-	-	5
Hiraiwa 2008 [15]	-	*	*	*	-	-	*	-	-	4

NOTE. REC: representativeness of the exposed cohort; SNEC: selection of the non-exposed cohort; AE: ascertainment of exposure; DO: demonstration that outcome of interest was not present at start of study; SC: study controls for age, sex; AF: study controls for any additional factors (chemoradiotherapy, curative resection); AO: assessment of outcome; FU: follow-up long enough (36M) for outcomes to occur; AFU: adequacy of follow-up of cohorts (≥90%).'*' means that the study is satisfied the item (high quality with no bias), and '-' means that the study is not satisfied the item (low quality with bias); Total: the number of high-quality items (no bias) in each study.

### Association between the presence of CTCs and tumor metastasis

**Figure 2:**
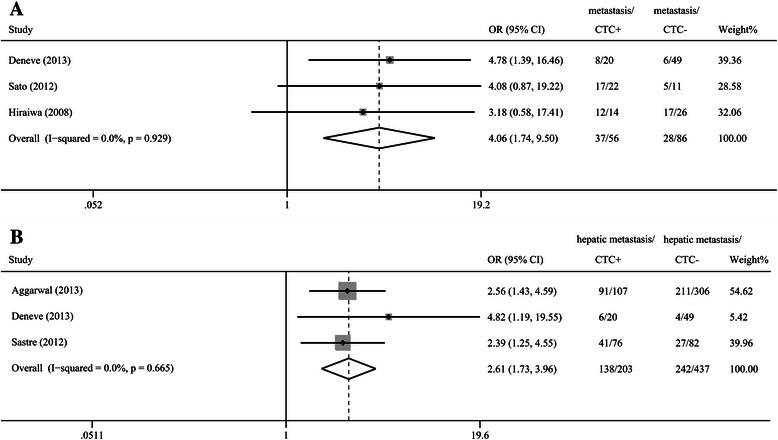
**Odds ratios summary for all kinds of tumor metastasis (A) and hepatic metastasis (B). A**: The estimated odds ratio (OR) was summarized for the relationship between all kinds of tumor metastasis and CTC detection. **B**: The OR was summarized for the relationship between hepatic metastasis and CTC detection.

Three studies reported the incidence of CTCs in metastasis-positive and -negative groups [13,15,29]: one study showed a statistically significant difference between the groups [13] and two studies showed a worse trend toward metastasis in patients with CTCs [15,29], although the differences were not statistically significant. A meta-analysis of all the relevant studies of metastasis showed a significantly higher incidence of CTCs in the metastasis-positive groups than in the metastasis-negative groups (OR = 4.06, 95% CI [1.74, 9.50], P < 0.01, I^2^ = 0%; Figure 2A). We also conducted a meta-analysis to investigate the association between hepatic metastasis, which is the most common type of metastasis in patients with CRC, and the detection of CTCs. The pooled results showed a significantly higher incidence of CTCs in the hepatic-metastasis-positive groups than in the hepatic-metastasis-negative groups (OR = 2.61, 95% CI [1.73, 3.96], P < 0.01, I^2^ = 0%; Figure 2B).

### Impact of CTCs on survival outcomes (OS and PFS)

**Figure 3:**
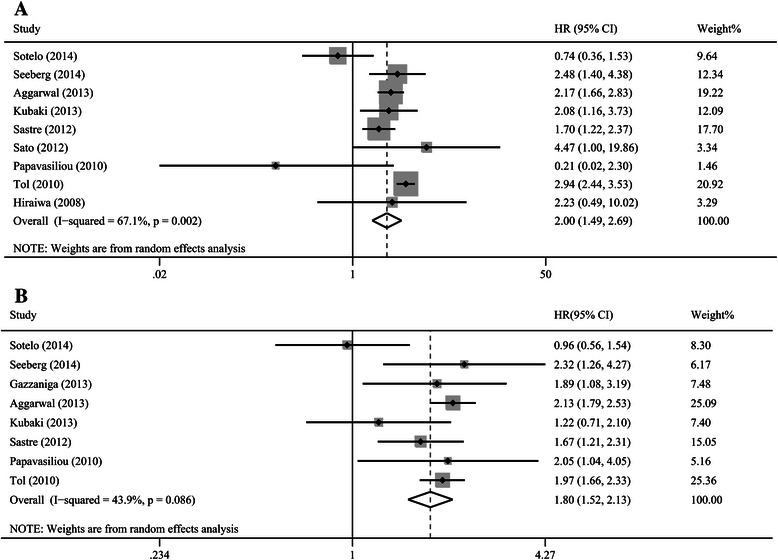
**Hazard ratios summary for overall survival (A) and progression-free survival (B). A**: The estimated hazard ratio (HR) was summarized for overall survival with CTC detection. **B**: The estimated HR was summarized for progression-free survival with CTC detection.

The HRs for OS and PFS were available in nine [14,15,24,25,27-31] and eight [14,24-28,30,31] studies, respectively. The pooled analysis revealed that the detection of CTCs with the CellSearch System in patients with CRC was associated with a worse OS (HR = 2.00, 95% CI [1.49, 2.69], P < 0.01, I^2^ = 67.1%; Figure 3A) and a worse PFS (HR = 1.80, 95% CI [1.52, 2.13], P < 0.01, I^2^ = 43.9%; Figure 3B). Sensitivity analyses confirmed the stability of our results, and indicated that our results were not obviously affected and dominated by any single study or different cut-off values.

In the subgroup analysis based on sampling time, a significant prognostic effect of CTC detection was confirmed in the analysis of studies that collected the samples at baseline (OS: HR = 1.78, 95% CI [1.34, 2.37], P < 0.01, I^2^ = 54.3%; PFS: HR = 1.55, 95% CI [1.35, 1.77], P < 0.01, I^2^ = 4.0%), as well as during-treatment (OS: HR = 3.02, 95% CI [2. 07, 4.40], P = 0.01, I^2^ = 61.6%; PFS: HR = 2.50, 95% CI [2.14, 2.92], P < 0.01, I^2^ = 0.0%). Four studies assessed the prognostic value of CTCs at various cut-off values [24-26,29]. Subgroup analyses based on cut-off values indicated that CTC^+^ at cut-off values of ≥1/7.5 ml (OS: HR = 2.94, 95% CI [0.51-17.01], P = 0.23; PFS: HR = 1.46, 95% CI [0.92-2.31], P = 0.11) and ≥2/7.5 ml (OS: HR = 1.67, 95% CI [0.84-3.31], P = 0.15; PFS: HR = 1.53, 95% CI [0.72-3.25], P = 0.27) tended to have an unfavorable prognosis, although statistical significance was not reached. And the result for cut-off values of ≥3/7.5 ml (OS: HR = 1.66, 95% CI [1.14-2.42], P < 0.01; PFS: HR = 1.79, 95% CI [1.52-2.11], P < 0.01) reached statistical significance.

The results of quality assessment of the included studies (Table 2) were summarized in Table 2. After excluding the study with lowest quality (NOS Score = 3) [30], our results still indicated that CTC^+^ group was associated with a worse OS and PFS in patients with CRC (OS:HR = 2.08, 95% CI [1.57, 2.75, P < 0.01, I^2^ = 65.6%; PFS:HR = 1.77, 95% CI [1.48, 2.13, P < 0.01, I^2^ = 51.7%).

### Objective response to adjuvant chemotherapy

**Figure 4:**
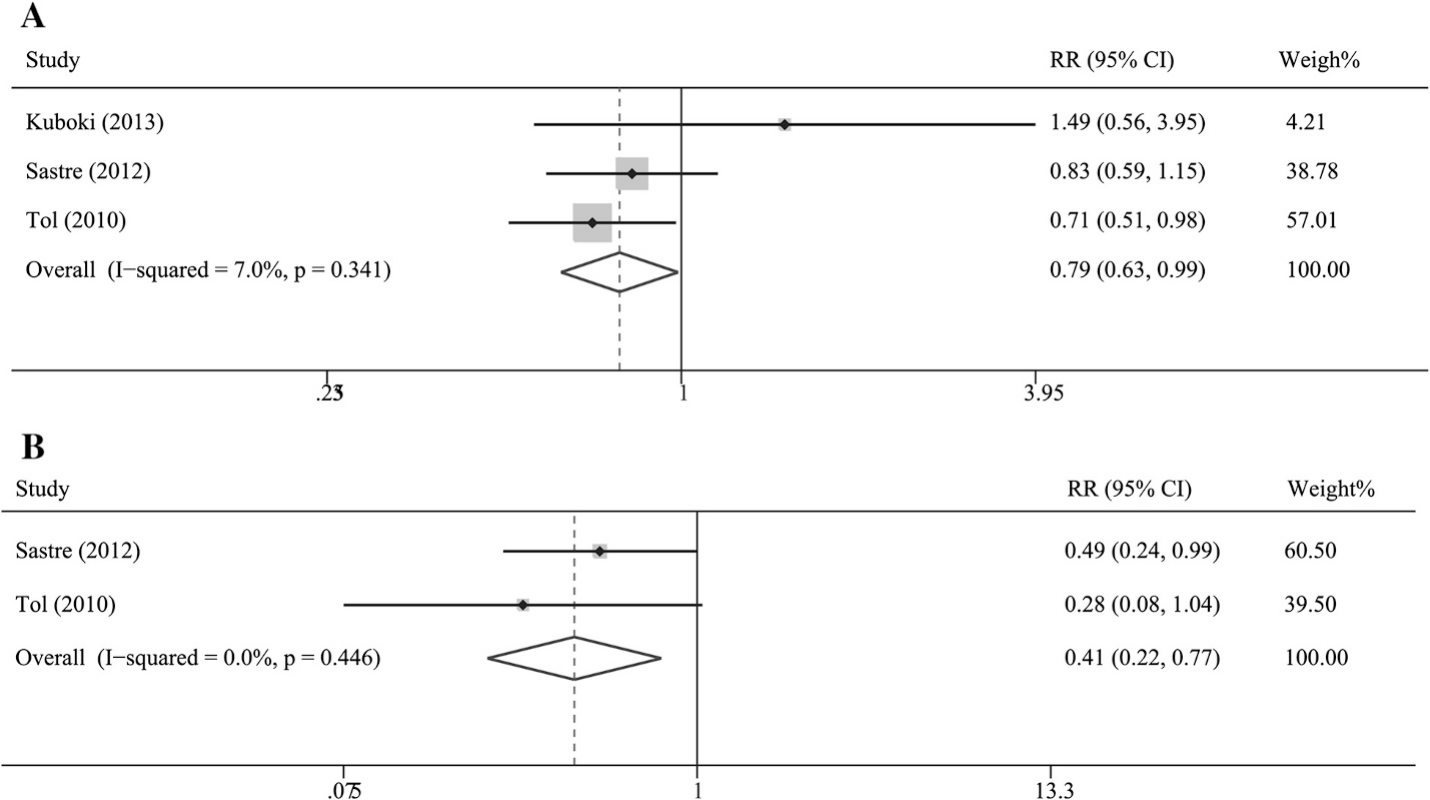
**Risk ratios summary for the correlation of tumor response and CTCs. A**: The estimated risk ratio (RR) was summarized for the correlation of tumor response with CTCs detected at baseline time. **B**: The estimated RR was summarized for the correlation of tumor response with CTCs detected at during-treatment time.

Only three studies assessed the correlation between CTCs and radiographic imaging results in patients receiving adjuvant chemotherapy [14,31,32]. The pooled results showed that the response rate for the CTC^+^ groups was significantly lower than that for the CTC^−^ groups at baseline and during treatment (baseline: 33%, 95% CI [27%, 39%] versus 39%, 95% CI [35%, 44%], RR = 0.79, 95% CI [0.63, 0.99], P = 0.04, I^2^ = 7.0%, Figure 4A; during treatment: 17%, 95% CI [6%, 28%] versus 46%, 95% CI [33%, 59%], RR = 0.41, 95% CI [0.22, 0.77], P = 0.01, I^2^ = 0.0%, Figure 4B).

When we assumed radiographic imaging to be the gold-standard diagnostic procedure, the sensitivity for the baseline CTC^+^ group in detecting no-response events (SD + PD) was 37% (95% CI [32%, 42%]) and the specificity was 70% (95% CI [63%, 76%]), and summary diagnostic OR was 1.47 (95% CI [1.03, 2.10]). Sensitivity for the during-treatment CTC^+^ group was 13% (95% CI [9%, 17%]) and the specificity was 96% (95% CI [92%, 98%]), and summary diagnostic OR was 3.89 (95% CI [1.71, 8.88]). For the imaging disease progression events (PD), sensitivity and specificity for the baseline CTC^+^ group were 26% (95% CI [14%, 40%]) and 70% (95% CI [65%, 75%]), and summary diagnostic OR was 0.75 (95% CI [0.34, 1.66]). Sensitivity and specificity for the during-treatment CTC^+^ group were 26% (95% CI [17%, 36%]) and 94% (95% CI [92%, 96%]), and summary diagnostic OR was 4.73 (95% CI [2.56, 8.73]).

### Assessment of heterogeneity and publication bias

**Figure 5:**
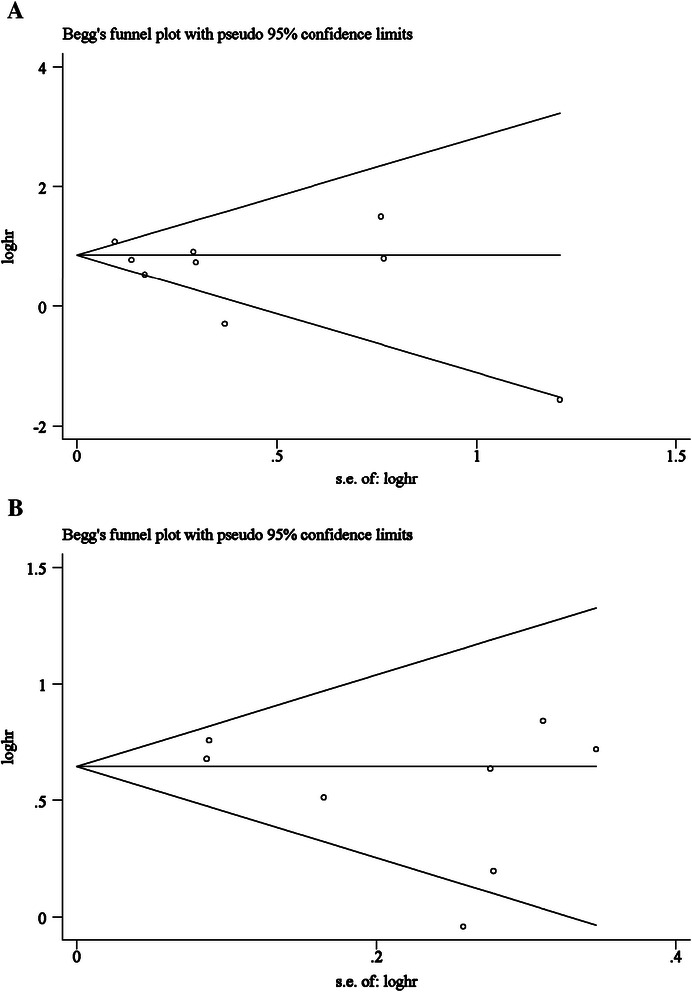
**Funnel plot analysis. A: overall survival; B: progression-free survival. A**: Funnel plot of the studies on overall survival. **B**: Funnel plot of the studies on progression-free survival.

Our meta-regression suggested that cut-off values, sampling time, sample size and publication year did not affect the estimated HRs for PFS and OS obviously. The results of meta-regression may be affected by limited number of studies. Moreover, Galbraith plot showed that the study by Sotelo et al. [24] may mainly contribute substantial heterogeneity to our meta-analysis. Potential publication bias was evaluated by Begg’s and Egger’s tests. There was no evidence of publication bias for the pooled analysis of OS (P_Begg_ = 0.60, P_Egger_ = 0.15) and PFS (P_Begg_ = 0.90, P_Egger_ = 0.18) (Figure 5).

## Discussion

In recent years, the prognosis of patients with CRC has improved remarkably after resection surgery combined with chemoradiotherapy [33,34]. However, the problems of metastasis, recurrence, and resistance to drugs are not yet resolved, and the causes and mechanisms of these phenomena have not been clarified [35]. Since CTCs were first identified in the PB of patients with CRC, the detection and study of CTCs have become a very topical issue for investigators worldwide [10]. A previous meta-analysis by Rahbari et al. reported that the presence of CTCs in the blood circulation of patients with CRC was an indicator of a poor prognosis [36], but that meta-analysis was limited by the presence of methodological heterogeneity because the enrolled studies used several different detection methods and were not stratified by method. The utility of CTC detection in the PB with the standardized CellSearch System has been demonstrated in several studies [37,38]. Deneve et al. reported that CTC detection in PB with the CellSearch System was convenient and correlates with tumor metastasis and prognosis [13]. However, Hiraiwa et al. and Kuboki et al. showed that the prognostic effects were not statistically significant [14,15]. Therefore, the prognostic role of CTC detection in the PB with the CellSearch System is still controversial.

This is the first comprehensive meta-analysis to determine the significance of CTC detection with the CellSearch System only. It suggests that patients with CTCs have poorer OS and PFS than those who lack CTCs, indicating that the clinical prognosis of patients with CRC is significantly associated with the CTCs detected in the PB with the CellSearch System. The absence of publication bias was confirmed with funnel plots (Figure 5).

To avoid the influence of temporal heterogeneity, a subgroup analysis was performed based on sampling time, and the estimated results for OS and PFS remained stable and were not markedly affected by sampling time. Our results indicated that CTCs detected not only at baseline but also during treatment could be considered a prognostic factor. These results are consistent with previous studies by Iinuma et al. and de Albuquerque et al., who used RT–PCR methods [7,39]. Uncertainties still remain about which sampling time (at baseline, during treatment, or at the completion of systemic treatment) could provide the most accurate prognostic information. The present study suggested that the association between prognosis and CTC detection was more pronounced and persuasive when the samples were collected during treatment than at baseline. The best explanation for this may be that the during-treatment CTC status provides relatively more information on survival outcomes than the baseline CTC status because the during-treatment CTC status combines the baseline CTC status and the tumor cells release during surgical manipulation [40]. Early-detected CTCs are not always associated with the survival outcome, because a portion of the early-detected CTCs can be inactivated and cleared by chemotherapeutic effects during chemotherapy, after which they will not affect the prognosis. Later samples could also contain additional CTCs that have been released from the primary tumor after changes in the tumor proliferative activity [41,42]. Therefore, later samples could more accurately reflect the CTC status by including CTC release, proliferation, apoptosis, and necrosis. Our results indicated that during-treatment sample collection was preferable to baseline collection in using CTCs to predict CRC outcomes.

Our result also showed a significantly higher incidence of CTCs in the hepatic-metastasis-positive groups than in the hepatic-metastasis-negative groups, which was consistent with a recent meta-analysis by Katsuno et al. [43] and supported the results of our meta-analysis of PFS. These results suggested that the liver could act as a suitable “soil” for CTCs and as an internal filter for tumor cells, which are shed at high concentrations from the primary tumor [44,45]. A possible explanation is that hepatic metastatic foci develop from micrometastatic foci formed by CTCs via hematogenous metastasis [4,46]. Furthermore, because the CellSearch System detects and quantifies CTCs based on the EpCAM protein on the CTCs, some investigators consider that EpCAM might play an important role in hepatic metastasis, cancer stemness, and the epithelial mesenchymal transition [47]. This hypothesis is supported by several studies that have reported that catumaxomab (anti-EpCAM × anti-CD3 antibodies) shows convincing therapeutic efficacy in patients with malignant tumors [48,49]. The high expression of the laminin receptor and the secretion of proteolytic enzymes by tumor cells also contribute to tumor migration and invasion [50,51].

Several studies have indicated that the presence of CTCs could be used to monitor the therapeutic effects of chemotherapy. In the studies we analyzed, only three [14,31,32] evaluated the correlation between CTCs and tumor responses on imaging according to the RECIST criteria. Our results indicated that CTCs detected at baseline or during treatment could predict the response to chemotherapy. Consequently, it might be appropriate to guide therapeutic decision-making on the basis of CTC counts, e.g., CTCs in the PB may be useful in identifying patients who could safely undertake prolonged treatment breaks from those who should resume therapy more rapidly [12]. The tailoring of targeted treatments could also be improved by the molecular analysis of epidermal growth factor receptor (EGFR) or Kirsten rat sarcoma viral oncogene homolog (KRAS genes) expression in CTCs, which have been identified as major biomarkers of resistance to anti-EGFR monoclonal antibodies (i.e., cetuximab) [52].

As with other methods, there is no consensus on the optimal cut-off value for CTCs in the PB for predicting the prognoses of patients with CRC. Although most studies used a cut-off value of CTCs ≥3/7.5 ml of blood and our results indicated that a cut-off value of CTCs ≥3/7.5 was available, an optimal cut-off value defining CTC positivity in patients with CRC is still not settled and several studies used various cut-off values to assess the clinical significance of CTCs [25,26]. The studies by Seeberg et al. and Gazzaniga et al. showed that cut-off value of CTCs ≥1/7.5 or ≥2/7.5 ml was also associated with poor prognosis in patients with CRC [25,26]. Moreover, our subgroup analyses suggested that CTC^+^ at cut-off values of CTCs ≥1/7.5 ml and≥≥ 2/7.5 ml also strongly tended to have an unfavorable prognosis. Thus, CRC patients with 1–2 CTC may be switched from the favorable prognostic group to the unfavorable prognostic group, deserving a more careful monitoring. Furthermore, it is unclear whether the cut-off values of CTCs are different between non-metastatic and metastatic CRC patients, considering the difference of cut-off values in non-metastatic and metastatic breast cancer (non-metastatic breast cancer: ≥1/7.5 ml; metastatic breast cancer: ≥5/7.5 ml) [53,54]. Thus, establishing a set of optimal cut-off values for CTC detection will require well-designed, large-scale multicenter studies.

As a semiautomated immunological technique, the CellSearch System has some obvious advantages relative to traditional ICC, including its easy operation, time effectiveness, and better CTC enrichment. Other advantages of the CellSearch System include its higher specificity and reproducibility than those of RT–PCR techniques. Moreover, CellSearch System could directly label the CTCs based on EpCAM, identify the viable versus nonviable CTCs, and visually acquire and quantify the CTCs [55]. In recent years, the use of the CellSearch System has been very widespread because it provides the further cytological analysis possible of CTCs, including the evaluation of the expression of chemotherapeutic or biologically therapeutic targets (e.g., EGFR and KRAS) [56].

Although our meta-analysis of studies that have used the CellSearch System reduced the heterogeneity caused by including studies based on other detection methods, there was still a considerable degree of heterogeneity in our meta-analysis. Specifically, in the pooled analysis of OS and PFS, heterogeneity was mainly caused by the study by Sotelo et al.[24]. Heterogeneity may also result from difference in patient characteristics (i.e., age, sex, or race), sampling time, or treatment regimens. The temporal and phenotypic heterogeneity of CTCs was also a source of heterogeneity. Differences in the experimental designs of the cohort studies also generated nonnegligible heterogeneity.

Several limitations must be addressed. First, as a retrospective study, our meta-analysis focused on the summary of published data from previous studies. Several studies did not provide HRs and we estimated them from the reported data. Second, there was considerable heterogeneity in our study. We addressed this heterogeneity by using a random-effects model to obtain more-conservative estimates if there was heterogeneity significant. It is well-known that the prognoses of patients with and without surgery are different. Therefore, most studies examined these two groups separately. However, Aggarwal et al., Kuboki et al., Tol et al., and Gazzaniga et al. did not report whether their patients underwent surgery [14,26,27,31]. We could not conduct a subgroup analysis, separating the patients into surgical and nonsurgical groups, in our meta-analysis because the number of studies was limited. The number of studies included here may have been insufficient to analyze the association between CTC detection and hepatic metastasis and the correlation between CTCs and tumor response to chemotherapy, which may have affected the internal and external validity of our results. Despite these limitations, our meta-analysis is the first study to evaluate the prognostic utility of CTCs detected with the CellSearch System in CRC patients.

## Conclusions

Our meta-analysis suggests that the detection of CTCs in the PB with the CellSearch System is a prognostic factor for patients with CRC. Future high-quality, well-designed multicenter studies are required to assess the clinical values and clinical utility of CTCs detected by CellSearch System in colorectal patients.

## Additional file

Additional file 1: **Search process.** The detailed description in the search process.

## Abbreviations

CIs: Confidence intervals
CR: Complete response
CRC: Colorectal cancer
CTCs: Circulating tumor cells
EGFR: Epidermal growth factor receptor
EpCAM: Epithelial cell adhesion molecule
HR: Hazard ratio
ICC: Immunocytochemistry
KRAS: Kirsten rat sarcoma viral oncogene homolog
mCRC: Metastatic CRC
NOS: Newcastle–Ottawa Scale
NR: Not reported
OR: Odds ratio OS, overall survival
PB: Peripheral blood
PD: Progressive disease
PFS: Progression-free survival
PR: Partial response
RECIST: Response evaluation criteria in solid tumors
RR: Risk ratio
RT–PCR: Transcription–polymerase chain reaction
SD: Stable disease

## References

[1] Jemal A, Bray F, Center MM, Ferlay J, Ward E, Forman D (2011). Global cancer statistics. CA Cancer J Clin.

[2] Seymour MT, Maughan TS, Ledermann JA, Topham C, James R, Gwyther SJ (2007). Different strategies of sequential and combination chemotherapy for patients with poor prognosis advanced colorectal cancer (MRC FOCUS): a randomised controlled trial. Lancet.

[3] Shah SA, Haddad R, Al-Sukhni W, Kim RD, Greig PD, Grant DR (2006). Surgical resection of hepatic and pulmonary metastases from colorectal carcinoma. J Am Coll Surg.

[4] Fidler IJ (2003). The pathogenesis of cancer metastasis: the ‘seed and soil’ hypothesis revisited. Nat Rev Cancer.

[5] Chen WS, Chung MY, Liu JH, Liu JM, Lin JK (2004). Impact of circulating free tumor cells in the peripheral blood of colorectal cancer patients during laparoscopic surgery. World J Surg.

[6] Sadahiro S, Suzuki T, Maeda Y, Yurimoto S, Yasuda S, Makuuchi H (2007). Detection of carcinoembryonic antigen messenger RNA-expressing cells in peripheral blood 7 days after curative surgery is a novel prognostic factor in colorectal cancer. Ann Surg Oncol.

[7] de Albuquerque A, Kubisch I, Stolzel U, Ernst D, Boese-Landgraf J, Breier G (2012). Prognostic and predictive value of circulating tumor cell analysis in colorectal cancer patients. J Transl Med.

[8] Wong SC, Ng SS, Cheung MT, Luk LY, Chan CM, Cheung AH (2011). Clinical significance of CDX2-positive circulating tumour cells in colorectal cancer patients. Br J Cancer.

[9] CELLSEARCH® Circulating Tumor Cell Kit (Epithelial) Instructions for Use. Janssen Diagnostics, LLC. 2014. https://www.cellsearchctc.com. Accessed 20 Apr 2014.

[10] Riethdorf S, Fritsche H, Muller V, Rau T, Schindlbeck C, Rack B (2007). Detection of circulating tumor cells in peripheral blood of patients with metastatic breast cancer: a validation study of the Cell Search system. Clin Cancer Res.

[11] Groot Koerkamp B, Rahbari NN, Buchler MW, Koch M, Weitz J (2013). Circulating tumor cells and prognosis of patients with resectable colorectal liver metastases or widespread metastatic colorectal cancer: a meta-analysis. Ann Surg Oncol.

[12] Cohen SJ, Punt CJ, Iannotti N, Saidman BH, Sabbath KD, Gabrail NY (2008). Relationship of circulating tumor cells to tumor response, progression-free survival, and overall survival in patients with metastatic colorectal cancer. J Clin Oncol.

[13] Deneve E, Riethdorf S, Ramos J, Nocca D, Coffy A, Daures JP (2013). Capture of viable circulating tumor cells in the liver of colorectal cancer patients. Clin Chem.

[14] Kuboki Y, Matsusaka S, Minowa S, Shibata H, Suenaga M, Shinozaki E (2013). Circulating tumor cell (CTC) count and epithelial growth factor receptor expression on CTCs as biomarkers for cetuximab efficacy in advanced colorectal cancer. Anticancer Res.

[15] Hiraiwa K, Takeuchi H, Hasegawa H, Saikawa Y, Suda K, Ando T (2008). Clinical significance of circulating tumor cells in blood from patients with gastrointestinal cancers. Ann Surg Oncol.

[16] Therasse P, Arbuck SG, Eisenhauer EA, Wanders J, Kaplan RS, Rubinstein L (2000). New guidelines to evaluate the response to treatment in solid tumors. European Organization for Research and Treatment of Cancer, National Cancer Institute of the United States, National Cancer Institute of Canada. J Natl Cancer Inst.

[17] Stang A (2010). Critical evaluation of the Newcastle-Ottawa scale for the assessment of the quality of nonrandomized studies in meta-analyses. Eur J Epidemiol.

[18] Panic N, Leoncini E, de Belvis G, Ricciardi W, Boccia S (2013). Evaluation of the endorsement of the preferred reporting items for systematic reviews and meta-analysis (PRISMA) statement on the quality of published systematic review and meta-analyses. PLoS One.

[19] Zamora J, Abraira V, Muriel A, Khan K, Coomarasamy A (2006). Meta-DiSc: a software for meta-analysis of test accuracy data. BMC Med Res Methodol.

[20] Tierney JF, Stewart LA, Ghersi D, Burdett S, Sydes MR (2007). Practical methods for incorporating summary time-to-event data into meta-analysis. Trials.

[21] Higgins JP, Thompson SG, Deeks JJ, Altman DG (2003). Measuring inconsistency in meta-analyses. BMJ.

[22] Egger M, Davey Smith G, Schneider M, Minder C (1997). Bias in meta-analysis detected by a simple, graphical test. BMJ.

[23] Begg CB, Mazumdar M (1994). Operating characteristics of a rank correlation test for publication bias. Biometrics.

[24] Sotelo MJ, Sastre J, Maestro ML, Veganzones S, Vieitez JM, Alonso V (2015). Role of circulating tumor cells as prognostic marker in resected stage III colorectal cancer. Ann Oncol.

[25] Seeberg LT, Waage A, Brunborg C, Hugenschmidt H, Renolen A, Stav I (2015). Circulating tumor cells in patients with colorectal liver metastasis predict impaired survival. Ann Surg.

[26] Gazzaniga P, Raimondi C, Gradilone A, Biondi Zoccai G, Nicolazzo C, Gandini O (2013). Circulating tumor cells in metastatic colorectal cancer: do we need an alternative cutoff?. J Cancer Res Clin Oncol.

[27] Aggarwal C, Meropol NJ, Punt CJ, Iannotti N, Saidman BH, Sabbath KD (2013). Relationship among circulating tumor cells, CEA and overall survival in patients with metastatic colorectal cancer. Annals Oncol.

[28] Sastre J, Maestro ML, Gomez-Espana A, Rivera F, Valladares M, Massuti B (2012). Circulating tumor cell count is a prognostic factor in metastatic colorectal cancer patients receiving first-line chemotherapy plus bevacizumab: a Spanish Cooperative Group for the Treatment of Digestive Tumors study. Oncologist.

[29] Sato N, Hayashi N, Imamura Y, Tanaka Y, Kinoshita K, Kurashige J (2012). Usefulness of transcription-reverse transcription concerted reaction method for detecting circulating tumor cells in patients with colorectal cancer. Ann Surg Oncol.

[30] Papavasiliou P, Fisher T, Kuhn J, Nemunaitis J, Lamont J (2010). Circulating tumor cells in patients undergoing surgery for hepatic metastases from colorectal cancer. Proc.

[31] Tol J, Koopman M, Miller MC, Tibbe A, Cats A, Creemers GJ (2010). Circulating tumour cells early predict progression-free and overall survival in advanced colorectal cancer patients treated with chemotherapy and targeted agents. Annals Oncol.

[32] Sastre J, Vidaurreta M, Gomez A, Rivera F, Massuti B, Lopez MR (2013). Prognostic value of the combination of circulating tumor cells plus kras in patients with metastatic colorectal cancer treated with chemotherapy plus bevacizumab. Clin Colorectal Cancer.

[33] Sobrero AF, Maurel J, Fehrenbacher L, Scheithauer W, Abubakr YA, Lutz MP (2008). EPIC: phase III trial of cetuximab plus irinotecan after fluoropyrimidine and oxaliplatin failure in patients with metastatic colorectal cancer. J Clin Oncol.

[34] Zinser-Sierra JW, Rodriguez-Ramirez S, Villalobos-Valencia R, Ramirez-Marquez M (2011). Use of bevacizumab in metastatic colorectal cancer: report from the Mexican opinion and analysis forum on colorectal cancer treatment with bevacizumab (September 2009). Drugs R D.

[35] Center MM, Jemal A, Smith RA, Ward E (2009). Worldwide variations in colorectal cancer. CA Cancer J Clin.

[36] Rahbari NN, Aigner M, Thorlund K, Mollberg N, Motschall E, Jensen K (2010). Meta-analysis shows that detection of circulating tumor cells indicates poor prognosis in patients with colorectal cancer. Gastroenterology.

[37] Yalcin S, Kilickap S, Portakal O, Arslan C, Hascelik G, Kutluk T (2010). Determination of circulating tumor cells for detection of colorectal cancer progression or recurrence. Hepatogastroenterology.

[38] Hayes DF, Cristofanilli M, Budd GT, Ellis MJ, Stopeck A, Miller MC (2006). Circulating tumor cells at each follow-up time point during therapy of metastatic breast cancer patients predict progression-free and overall survival. Clin Cancer Res.

[39] Iinuma H, Watanabe T, Mimori K, Adachi M, Hayashi N, Tamura J (2011). Clinical significance of circulating tumor cells, including cancer stem-like cells, in peripheral blood for recurrence and prognosis in patients with Dukes’ stage B and C colorectal cancer. J Clin Oncol.

[40] Weitz J, Kienle P, Lacroix J, Willeke F, Benner A, Lehnert T (1998). Dissemination of tumor cells in patients undergoing surgery for colorectal cancer. Clin Cancer Res.

[41] Molnar B, Sipos F, Galamb O, Tulassay Z (2003). Molecular detection of circulating cancer cells. Role in diagnosis, prognosis and follow-up of colon cancer patients. Dig Dis.

[42] Lim SH, Becker TM, Chua W, Caixeiro NJ, Ng WL, Kienzle N (2014). Circulating tumour cells and circulating free nucleic acid as prognostic and predictive biomarkers in colorectal cancer. Cancer Lett.

[43] Katsuno H, Zacharakis E, Aziz O, Rao C, Deeba S, Paraskeva P (2008). Does the presence of circulating tumor cells in the venous drainage of curative colorectal cancer resections determine prognosis? A meta-analysis. Ann Surg Oncol.

[44] Colombano SP, Reese PA (1980). The cascade theory of metastatic spread: are there generalizing sites?. Cancer.

[45] Taniguchi T, Makino M, Suzuki K, Kaibara N (2000). Prognostic significance of reverse transcriptase-polymerase chain reaction measurement of carcinoembryonic antigen mRNA levels in tumor drainage blood and peripheral blood of patients with colorectal carcinoma. Cancer.

[46] Stoecklein NH, Hosch SB, Bezler M, Stern F, Hartmann CH, Vay C (2008). Direct genetic analysis of single disseminated cancer cells for prediction of outcome and therapy selection in esophageal cancer. Cancer Cell.

[47] Dieter SM, Ball CR, Hoffmann CM, Nowrouzi A, Herbst F, Zavidij O (2011). Distinct types of tumor-initiating cells form human colon cancer tumors and metastases. Cell Stem Cell.

[48] Heiss MM, Strohlein MA, Jager M, Kimmig R, Burges A, Schoberth A (2005). Immunotherapy of malignant ascites with trifunctional antibodies. Int J Cancer.

[49] Burges A, Wimberger P, Kumper C, Gorbounova V, Sommer H, Schmalfeldt B (2007). Effective relief of malignant ascites in patients with advanced ovarian cancer by a trifunctional anti-EpCAM x anti-CD3 antibody: a phase I/II study. Clin Cancer Res.

[50] Gotzmann J, Mikula M, Eger A, Schulte-Hermann R, Foisner R, Beug H (2004). Molecular aspects of epithelial cell plasticity: implications for local tumor invasion and metastasis. Mutat Res.

[51] Aznavoorian S, Murphy AN, Stetler-Stevenson WG, Liotta LA (1993). Molecular aspects of tumor cell invasion and metastasis. Cancer.

[52] Raimondi C, Nicolazzo C, Gradilone A, Giannini G, De Falco E, Chimenti I (2014). Circulating tumor cells: Exploring intratumor heterogeneity of colorectal cancer. Cancer Biol Ther.

[53] Cristofanilli M, Budd GT, Ellis MJ, Stopeck A, Matera J, Miller MC (2004). Circulating tumor cells, disease progression, and survival in metastatic breast cancer. N Engl J Med.

[54] Lucci A, Hall CS, Lodhi AK, Bhattacharyya A, Anderson AE, Xiao L (2012). Circulating tumour cells in non-metastatic breast cancer: a prospective study. Lancet Oncol.

[55] Thorsteinsson M, Jess P (2011). The clinical significance of circulating tumor cells in non-metastatic colorectal cancer–a review. European J Surg Oncol.

[56] Torino F, Bonmassar E, Bonmassar L, De Vecchis L, Barnabei A, Zuppi C (2013). Circulating tumor cells in colorectal cancer patients. Cancer Treat Rev.

