# Supplementary material for: Meta-analysis of the prognostic value of circulating tumor cells detected with the CellSearch System in colorectal cancer
Source: BMC Cancer. 2015 Mar 30;15:202. doi: 10.1186/s12885-015-1218-9 (PMC4389311; doi:10.1186/s12885-015-1218-9)
Supplement: Additional file 1: — Search process. The detailed description in the search process. [file 12885_2015_1218_MOESM1_ESM.pdf]

Search Process (up to December, 2014)

1. Databases, results and search strategies

PubMed: 60 studies

Search strategies: (circulating tumor cells OR micrometastasis OR disseminated tumor cells OR isolated tumor cells OR occult tumor cells) AND CellSearch AND (colorectal cancer OR rectal cancer OR colon cancer)

Embase: 106 studies

Search strategies: (circulating tumor cells OR micrometastasis OR disseminated tumor cells OR isolated tumor cells OR occult tumor cells).af. AND (CellSearch).af. AND (colorectal cancer OR rectal cancer OR colon cancer).af.

Web of Science: 110 studies

Search strategies: (TS=circulating tumor cells OR TS=micrometastasis OR TS=disseminated tumor cells OR TS=isolated tumor cells OR TS=occult tumor cells) AND TS=CellSearch AND (TS=colorectal cancer OR TS=rectal cancer OR TS=colon cancer)

Cochrane Library: 3 studies

Search strategies: (circulating tumor cells OR micrometastasis OR disseminated tumor cells OR isolated tumor cells OR occult tumor cells) AND CellSearch AND (colorectal cancer OR rectal cancer OR colon cancer)

Manual search for reference lists: 13 studies

2. Excluded Studies by screening title and abstract

(1): 40 studies with other cancer:

1. Zovato S, Opocher G, Rossi E, Indraccolo S, Amadori A, Pastorelli D, Lombardi G, Anglani M, Zamarchi R: **Predictive value and biologic significance of circulating tumor cells (CTC) in sporadic and von hippel lindau (VHL) renal cancer**. In., vol. 7, 2-3 edn: Elsevier Ltd; 2009: 436-437.
2. Yang JD, Campion MB, Chaiteerakij R, Mettler TA, Giama NH, Zhang X, Hu C, Rud J, Halling KC, Roberts LR: **Circulating tumor cells are associated with more advanced disease in cholangiocarcinoma**. In., vol. 144, 5 SUPPL. 1 edn: W.B. Saunders; 2013: S957.
3. Yang JD, Campion MB, Chaiteerakij R, Mettler TA, Giama NH, Campion VL, Halling KC, Roberts LR: **Circulating tumor cells in cholangiocarcinoma**. In., vol. 140, 5 SUPPL. 1 edn: W.B. Saunders; 2011: S921.
4. Wu W, Woo J, Melnikova VO, Pace M, Gupta V, Jouravleva E, Anderes KL, Ackermann EJ, Kwoh TJ, Davis DW: **Expression of eIF4E relevant biomarkers detected in circulating tumor cells from metastatic NSCLC and prostate cancer patients using antibody-independent ApoStream technology**. In., vol. 30, 15 SUPPL. 1 edn: American Society of Clinical Oncology; 2012.
5. Van der Auwera I, Elst HJ, Van Laere SJ, Maes H, Huget P, van Dam P, Van Marck EA, Vermeulen PB, Dirix LY: **The presence of circulating total DNA and methylated genes is associated with circulating tumour cells in blood from breast cancer patients**. *British journal of cancer* 2009, **100**(8):1277-1286.
6. Tanaka F, Yoneda K, Kondo N, Hashimoto M, Takuwa T, Matsumoto S, Okumura Y, Rahman S, Tsubota N, Tsujimura T *et al*: **Circulating Tumor Cell as a Diagnostic Marker in Primary Lung Cancer**. *Clinical Cancer Research* 2009, **15**(22):6980-6986.
7. Smerage JB, Budd GT, Doyle GV, Brown M, Paoletti C, Muniz M, Miller MC, Repollet MI, Chianese DA, Connelly MC *et al*: **Monitoring apoptosis and Bcl-2 on circulating tumor cells in patients with metastatic breast cancer**. *Molecular oncology* 2013, **7**(3):680-692.
8. Sieuwerts AM, Kraan J, Bolt-De Vries J, Van Der Spoel P, Elstrodt F, Smid M, Timmermans M, Mostert B, Schutte M, Martens JWM *et al*: **Response: Re: Anti-Epithelial Cell Adhesion Molecule Antibodies and the Detection of Circulating Normal-Like Breast Tumor Cells**. *Journal of the National Cancer Institute* 2009, **101**(12):896-897.
9. Sclafani F, Smyth E, Cunningham D, Chau I, Turner A, Watkins D: **A pilot study assessing the incidence and clinical significance of circulating tumor**

cells in esophagogastric cancers. *Clinical colorectal cancer* 2014, **13**(2):94-99.

10. Schneck H, Blassl C, Meier-Stiegen F, Neves RP, Janni W, Fehm T, Neubauer H: **Analysing the mutational status of PIK3CA in circulating tumor cells from metastatic breast cancer patients**. *Molecular oncology* 2013, **7**(5):976-986.
11. Scher HI, Morris MJ, Basch E, Heller G: **End Points and Outcomes in Castration-Resistant Prostate Cancer: From Clinical Trials to Clinical Practice**. *Journal Of Clinical Oncology* 2011, **29**(27):3695-3704.
12. Samoila A, Wan M, Anand A, Chuang T, Scher HI, Fleisher M, Danila DC: **A pilot study comparing the sensitivity of detecting prostate associated transcripts by AdnaTest prostate assay to CellSearch in whole blood from patients with castration resistant prostate cancer**. In., vol. 14, 6 edn: Elsevier; 2012: 709.
13. Poveda A, Kaye SB, McCormack R, Wang S, Parekh T, Ricci D, Lebedinsky CA, Tercero JC, Zintl P, Monk BJ: **Circulating tumor cells predict progression free survival and overall survival in patients with relapsed/recurrent advanced ovarian cancer**. *Gynecologic oncology* 2011, **122**(3):567-572.
14. Park Y, Kitahara T, Urita T, Yoshida Y, Kato R: **Expected clinical applications of circulating tumor cells in breast cancer**. *World journal of clinical oncology* 2011, **2**(8):303-310.
15. Park S, Ang RR, Duffy SP, Bazov J, Chi KN, Black PC, Ma H: **Morphological Differences between Circulating Tumor Cells from Prostate Cancer Patients and Cultured Prostate Cancer Cells**. *PloS one* 2014, **9**(1).
16. Okumura Y, Tanaka F, Yoneda K, Hashimoto M, Takuwa T, Kondo N, Hasegawa S: **Circulating Tumor Cells in Pulmonary Venous Blood of Primary Lung Cancer Patients**. *Annals Of Thoracic Surgery* 2009, **87**(6):1669-1675.
17. O'Flaherty JD, Gray S, Richard D, Fennell D, O'Leary JJ, Blackhall FH, O'Byrne KJ: **Circulating tumour cells, their role in metastasis and their clinical utility in lung cancer**. *Lung cancer* 2012, **76**(1):19-25.
18. Negin BP, Meropol NJ, Alpaugh RK, Ruth K, McAleer C, Halbherr T, Bingham C, Fittipaldi P, Cohen SJ: **Characterization and prognostic significance of circulating tumor cells in the peripheral blood of patients with metastatic pancreatic cancer**. In., vol. 28, 15 SUPPL. 1 edn: American Society of Clinical Oncology; 2010.
19. Naito T, Tanaka F, Ono A, Yoneda K, Takahashi T, Murakami H, Nakamura Y, Tsuya A, Kenmotsu H, Shukuya T *et al*: **Prognostic Impact of Circulating Tumor Cells in Patients with Small Cell Lung Cancer**. *Journal Of Thoracic Oncology* 2012, **7**(3):512-519.
20. Murez T, Droupy S, Rebillard X, Alix-Panabieres C: **[Circulating tumor cells and advanced prostate cancer]**. *Bulletin du cancer* 2012, **99 Suppl 1**:S4-15.

21. Mueller V, Riethdorf S, Rack B, Janni W, Fasching PA, Solomayer E, Aktas B, Kasimir-Bauer S, Zeitz J, Pantel K *et al*: **Prospective evaluation of serum tissue inhibitor of metalloproteinase 1 and carbonic anhydrase IX in correlation to circulating tumor cells in patients with metastatic breast cancer**. *Breast Cancer Research* 2011, **13**(4).
22. Melnikova VO, Davis DW, Mittelman D, Zhang Y, Holz GS, Doshi GK, Cen P, Amato RJ: **Development of a new, sensitive assay for circulating tumor cell detection in prostate cancer**. In., vol. 28, 15 SUPPL. 1 edn: American Society of Clinical Oncology; 2010.
23. Lorente D, Mateo J, de Bono JS: **Molecular characterization and clinical utility of circulating tumor cells in the treatment of prostate cancer**. *American Society of Clinical Oncology educational book / ASCO American Society of Clinical Oncology Meeting* 2014:e197-203.
24. Loh J, Jovanovic L, Lehman M, Capp A, Pryor D, Harris M, Nelson C, Martin J: **Circulating tumor cell detection in high-risk non-metastatic prostate cancer**. *Journal of cancer research and clinical oncology* 2014, **140**(12):2157-2162.
25. Le Rhun E, Massin F, Tu Q, Bonneterre J, Bittencourt Mde C, Faure GC: **Development of a new method for identification and quantification in cerebrospinal fluid of malignant cells from breast carcinoma leptomeningeal metastasis**. *BMC clinical pathology* 2012, **12**:21.
26. Krebs MG, Hou J-M, Sloane R, Lancashire L, Priest L, Nonaka D, Ward TH, Backen A, Clack G, Hughes A *et al*: **Analysis of Circulating Tumor Cells in Patients with Non-small Cell Lung Cancer Using Epithelial Marker-Dependent and -Independent Approaches**. *Journal Of Thoracic Oncology* 2012, **7**(2):306-315.
27. Kindelberger DW, Matulonis U: **The utility of circulating tumor cells in monitoring patients with recurrent ovarian cancer**. In., vol. 90: Nature Publishing Group; 2010: 250A.
28. Kaifi JT, Kunkel M, Allen JE, Dicker DT, Das A, Gusani NJ, Reed MF, Kimchi ET, Staveley-O'Carroll KF, El-Deiry WS: **Presence of circulating tumor cells in patients with resectable colorectal cancer lung metastases is a potential indicator for extrathoracic disease**. In., vol. 179, 2 edn: Academic Press Inc.; 2013.
29. Hosokawa M, Kenmotsu H, Koh Y, Yoshino T, Yoshikawa T, Naito T, Takahashi T, Murakami H, Nakamura Y, Tsuya A *et al*: **Size-based isolation of circulating tumor cells in lung cancer patients using a microcavity array system**. *PloS one* 2013, **8**(6):e67466.
30. Hashimoto M, Tanaka F, Yoneda K, Takuwa T, Matsumoto S, Okumura Y, Kondo N, Hasegawa S, Tsujimura T, Nakano T: **Circulating tumor cells (CTCs) in patients who underwent lung resection for lung metastases**. In., vol. 9: European Association for Cardio-Thoracic Surgery; 2009: S29-S30.
31. Gurtowska N, Bajek A, Olkowska J, Drewa T: **Identification of circulating tumor cells as a promising method of genitourinary cancer diagnosis**.

Postepy higieny i medycyny doswiadczalnej 2012, **66**:983-990.
32. Gazzaniga P, Gradilone A, De Berardinis E, Nicolazzo C, Petracca A, Farcomeni A, Busetto GM, Gentile V, Frati L, Cortesi E: **Prognostic value of circulating tumor cells in non muscle invasive bladder cancer: A CellSearch analysis**. *European Urology Supplements* 2012, **11**(1):E900-U893.
33. Fornvik D, Andersson I, Dustler M, Ehrnstrom R, Ryden L, Tingberg A, Zackrisson S, Aaltonen K: **No evidence for shedding of circulating tumor cells to the peripheral venous blood as a result of mammographic breast compression**. *Breast cancer research and treatment* 2013, **141**(2):187-195.
34. Connelly M, Wang Y, Doyle GV, Terstappen L, McCormack R: **Re: Anti-Epithelial Cell Adhesion Molecule Antibodies and the Detection of Circulating Normal-Like Breast Tumor Cells**. *Journal of the National Cancer Institute* 2009, **101**(12):895-895.
35. Blonski M, Wittwer B, Faure G, Simon L, De Carvalho Bittencourt M, Tu Q, Larrieu D, Le Rhun E, Taillandier L: **Identification and quantification of CSF malignanT-cells by the CellSearch technology in patients with lung leptomeningeal metastasis**. In., vol. 21: Blackwell Publishing Ltd; 2014: 45.
36. Blonski M, Wittwer B, Faure G, Simon L, De Carvalho Bittencourt M, Tu Q, Larrieu D, Le Rhun E, Taillandier L: **Identification and quantification of CSF malignant cells by the CellSearch technology in patients with lung leptomeningeal metastasis**. In., vol. 261: D. Steinkopff-Verlag; 2014: S34.
37. Bidard FC, Mathiot C, Degeorges A, Etienne-Grimaldi MC, Delva R, Pivot X, Veyret C, Bergougnoux L, de Cremoux P, Milano G *et al*: **Clinical value of circulating endothelial cells and circulating tumor cells in metastatic breast cancer patients treated first line with bevacizumab and chemotherapy**. *Annals Of Oncology* 2010, **21**(9):1765-1771.
38. Alemar J, Schuur ER: **Progress in using circulating tumor cell information to improve metastatic breast cancer therapy**. In. United States: Hindawi Publishing Corporation (410 Park Avenue, 15th Floor, 287 pmb, New York NY 10022, United States); 2013.
39. Gross S, Foulk B, Patel J, Connelly M, Mata M: **Automated enumeration and characterization of circulating multiple myeloma cells in blood**. In., vol. 118, 21 edn: American Society of Hematology; 2011.
40. Truini A, Alama A, Dal Bello MG, Coco S, Vanni I, Rijavec E, Genova C, Barletta G, Biello F, Grossi F: **Clinical Applications of Circulating Tumor Cells in Lung Cancer Patients by CellSearch System**. *Frontiers in oncology* 2014, **4**:242.

(2): 71 studies not relevant

1. Zhou L, Yang Z, Baker M, Dicker DT, El-Deiry WS: **Multispectral imaging of mismatch repair proteins in colorectal cancers and tumor cells isolated from blood**. In., vol. 72, 8 SUPPL. 1 edn: American Association for Cancer

Research Inc.; 2012.
2. Zhe X, Cher ML, Bonfil RD: **Circulating tumor cells: Finding the needle in the haystack**. In., vol. 1, 6 edn. United States: E-Century Publishing Corporation (40 White Oaks Lane, Madison WI 53711, United States); 2011: 740-751.
3. Watanabe M, Uehara Y, Yamashita N, Fujimura Y, Nishio K, Suzuki J, Koizumi F, Koh Y: **Evaluation of a novel flow cytometry-based system for the detection of circulating tumor cells**. In., vol. 72, 8 SUPPL. 1 edn: American Association for Cancer Research Inc.; 2012.
4. Watanabe M, Uehara Y, Yamashita N, Fujimura Y, Nishio K, Sawada T, Takeda K, Koizumi F, Koh Y: **Multicolor detection of rare tumor cells in blood using a novel flow cytometry-based system**. *Cytometry Part A : the journal of the International Society for Analytical Cytology* 2014, **85**(3):206-213.
5. Watanabe M, Serizawa M, Sawada T, Takeda K, Takahashi T, Yamamoto N, Koizumi F, Koh Y: **A novel flow cytometry-based cell capture platform for the detection, capture and molecular characterization of rare tumor cells in blood**. *Journal of translational medicine* 2014, **12**.
6. Wang LH, Pfister TD, Parchment RE, Kummar S, Rubinstein L, Evrard YA, Gutierrez ME, Murgo AJ, Tomaszewski JE, Doroshow JH *et al*: **Monitoring drug-induced H2AX as a pharmacodynamic biomarker in individual circulating tumor cells**. In., vol. 16, 3 edn. United States: American Association for Cancer Research Inc. (615 Chestnut Street, 17th Floor, Philadelphia PA 19106-3483, United States); 2010: 1073-1084.
7. Sun YF, Yang XR, Zhou J, Qiu SJ, Fan J, Xu Y: **Circulating tumor cells: Advances in detection methods, biological issues, and clinical relevance**. In., vol. 137, 8 edn. Germany: Springer Verlag (Tiergartenstrasse 17, Heidelberg D-69121, Germany); 2011: 1151-1173.
8. Sua Villegas LF, Silva Perez NM, Vidaurreta Lazaro M, Maestro de las Casas ML, Rafael Fernandez S, Veganzones de Castro S: **Current and future techniques for quantifying circulating tumor cells: Importance in solid epithelial tumors**. In., vol. 4, 3 edn. Spain: Ediciones Doyma, S.L. (Travesera de Gracia 17-21, Barcelona 08021, Spain); 2011: 163-169.
9. Shahneh FZ: **Sensitive antibody-based CTCs detection from peripheral blood**. In., vol. 22, 1-2 edn. Netherlands: IOS Press (Nieuwe Hemweg 6B, Amsterdam 1013 BG, Netherlands); 2013: 51-54.
10. Seronie-Vivien S: **[Circulating tumor cells: a new challenge for laboratory medicine]**. *Annales de biologie clinique* 2014, **72**(2):153-177.
11. Scholtens TM, Schreuder F, Ligthart ST, Swennenhuis JF, Greve J, Terstappen LWMM: **Automated identification of circulating tumor cells by image cytometry**. *Cytometry Part A* 2012, **81A**(2):138-148.
12. Rossi E, Basso U, Celadin R, Zilio F, Pucciarelli S, Aieta M, Barile C, Sava T, Bonciarelli G, Tumolo S *et al*: **M30 neoepitope expression in epithelial cancer: Quantification of apoptosis in circulating tumor cells by**

CellSearch analysis. In., vol. 16, 21 edn. United States: American Association for Cancer Research Inc. (615 Chestnut Street, 17th Floor, Philadelphia PA 19106-3483, United States); 2010: 5233-5243.

13. Raimondi C, Gradilone A, Naso G, Cortesi N, Gazzaniga P: **Clinical utility of circulating tumor cell counting through CellSearch (R): the dilemma of a concept suspended in Limbo**. *OncoTargets and therapy* 2014, **7**:619-625.
14. Peters KL, Allen JE, Dicker DT, Das A, Joudeh J, El-Deiry WS: **CTC profiling: Integration of biomarker analysis with current Veridex CellSearch technology**. In., vol. 71, 8 SUPPL. 1 edn: American Association for Cancer Research Inc.; 2011.
15. Peeters DJE, De Laere B, Van den Eynden GG, Van Laere SJ, Rothe F, Ignatiadis M, Sieuwerts AM, Lambrechts D, Rutten A, van Dam PA *et al*: **Semiautomated isolation and molecular characterisation of single or highly purified tumour cells from CellSearch enriched blood samples using dielectrophoretic cell sorting**. *British journal of cancer* 2013, **108**(6):1358-1367.
16. Paterlini-Brechot P, Benali NL: **Circulating tumor cells (CTC) detection: Clinical impact and future directions**. In., vol. 253, 2 edn. Ireland: Elsevier Ireland Ltd; 2007: 180-204.
17. Parkinson DR, Dracopoli N, Petty BG, Compton C, Cristofanilli M, Deisseroth A, Hayes DF, Kapke G, Kumar P, Lee JSH *et al*: **Considerations in the development of circulating tumor cell technology for clinical use**. *Journal of translational medicine* 2012, **10**.
18. Pantel K, Deneve E, Nocca D, Coffy A, Vendrell JP, Maudelonde T, Riethdorf S, Alix-Panabieres C: **Circulating epithelial cells in patients with benign colon diseases**. *Clinical chemistry* 2012, **58**(5):936-940.
19. Pace M, Melnikova VO, Brandl K, Kahn L, Woo J, Davis DW: **BRAFV600E mutation analysis in circulating tumor cells**. In., vol. 71, 8 SUPPL. 1 edn: American Association for Cancer Research Inc.; 2011.
20. Ozkumur E, Shah AM, Ciciliano JC, Emmink BL, Miyamoto DT, Brachtel E, Yu M, Chen PI, Morgan B, Trautwein J *et al*: **Inertial focusing for tumor antigen-dependent and -independent sorting of rare circulating tumor cells**. In., vol. 5, 179 edn. United States: American Association for the Advancement of Science (1200 New York Avenue NW, Washington DC 20005, United States); 2013.
21. Negin BP, Cohen SJ: **Circulating tumor cells in colorectal cancer: Past, present, and future challenges**. In., vol. 11, 1-2 edn. United States: Springer New York (233 Springer Street, New York NY 10013-1578, United States); 2010: 1-13.
22. Mikulova V, Kolostova K, Zima T: **Methods for Detection of Circulating Tumour Cells and Their Clinical Value in Cancer Patients**. *Folia Biologica* 2011, **57**(4):151-161.
23. Melnikova VO, Liu W, Zhang Y, Hong DS, Davis DW: **Development of a new, highly sensitive assay for circulating tumor cell (CTC) detection**

based on the CellSearch (R) CTC Profile Kit enrichment and laser scanning cytometry analysis. *Proceedings of the American Association for Cancer Research Annual Meeting* 2010, **51**:652-653.

24. Mavroudis D: **Circulating cancer cells**. In., vol. 21, SUPPL. 7 edn. United Kingdom: Oxford University Press (Great Clarendon Street, Oxford OX2 6DP, United Kingdom); 2010: vii95-vii100.
25. Matsusaka S, Kozuka M, Takagi H, Ito H, Minowa S, Hirai M, Hatake K: **A novel detection strategy for living circulating tumor cells using 5-aminolevulinic acid**. In., vol. 355, 1 edn. Ireland: Elsevier Ireland Ltd; 2014: 113-120.
26. Ma X, Xiao Z, Li X, Wang F, Zhang J, Zhou R, Wang J, Liu L: **Prognostic role of circulating tumor cells and disseminated tumor cells in patients with prostate cancer: a systematic review and meta-analysis**. *Tumor Biology* 2014, **35**(6):5551-5560.
27. Lowes LE, Hedley BD, Keeney M, Allan AL: **Adaptation of semiautomated circulating tumor cell (CTC) assays for clinical and preclinical research applications**. *Journal of visualized experiments : JoVE* 2014(84):e51248.
28. Lowes LE, Hedley BD, Keeney M, Allan AL: **User-defined protein marker assay development for characterization of circulating tumor cells using the CellSearch(R) system**. *Cytometry Part A : the journal of the International Society for Analytical Cytology* 2012, **81**(11):983-995.
29. Lowes LE, Goodale D, Keeney M, Allan AL: **Image Cytometry Analysis of Circulating Tumor Cells**. In: *Recent Advances In Cytometry, Part A: Instrumentation, Methods, Fifth Edition. Volume 102*, edn.; 2011: 261-290.
30. Lopez-Riquelme N, Minguela A, Villar-Permuy F, Ciprian D, Castillejo A, Alvarez-Lopez M-R, Soto J-L: **Imaging cytometry for counting circulating tumor cells: comparative analysis of the CellSearch vs ImageStream systems**. *Apmis* 2013, **121**(12):1139-1143.
31. Lianidou ES: **Circulating tumor cells-new challenges ahead**. In., vol. 58, 5 edn. United States: American Association for Clinical Chemistry Inc. (2101 L Street NW, Suite 202, Washington DC 20037-1526, United States); 2012: 805-807.
32. Le Rhun E, Tu Q, De Carvalho Bittencourt M, Farre I, Mortier L, Cai H, Kohler C, Faure GC: **Detection and quantification of CSF malignant cells by the CellSearch technology in patients with melanoma leptomeningeal metastasis**. *Medical oncology (Northwood, London, England)* 2013, **30**(2):538.
33. Le Rhun E, Tu Q, Bittencourt MDC, Farre I, Mortier L, Cai H, Kohler C, Faure GC: **Detection and quantification of CSF malignant cells by the CellSearch (R) technology in patients with melanoma leptomeningeal metastasis**. *Medical Oncology* 2013, **30**(2).
34. Kurbacher CM, Schmidt ME, Arenz PN, Nagel WJ, Stark R, Hanitzsch H, Kurbacher JA: **Circulating Tumour Cells: a Valuable New Tool to Monitor the Clinical Course of Patients With Epithelial Neoplasms in the Routine**

Setting. *European journal of cancer* 2011, **47**:S177-S177.

35. Krebs MG, Metcalf RL, Carter L, Brady G, Blackhall FH, Dive C: **Molecular analysis of circulating tumour cells-biology and biomarkers**. *Nature Reviews Clinical Oncology* 2014, **11**(3):129-144.
36. Komine K, Inoue M, Otsuka K, Fukuda K, Nanjo H, Shibata H: **Utility of Measuring Circulating Tumor Cell Counts to Assess the Efficacy of Treatment for Carcinomas of Unknown Primary Origin**. *Anticancer research* 2014, **34**(6):3165-3168.
37. King JD, Casavant BP, Lang JM: **Rapid translation of circulating tumor cell biomarkers into clinical practice: technology development, clinical needs and regulatory requirements**. *Lab on a chip* 2014, **14**(1):24-31.
38. Kasimir-Bauer S: **Circulating tumor cells as markers for cancer risk assessment and treatment monitoring**. In., vol. 13, 4 edn. New Zealand: Adis International Ltd (41 Centorian Drive, Private Bag 65901, Mairangi Bay, Auckland 10 1311, New Zealand); 2009: 209-215.
39. Joosse SA, Hannemann J, Alix-Panabieres C, Deneve E, Beneken C, Nieuwland M, Kerkhoven RM, Ramos J, Wikman H, Riethdorf L *et al*: **High-resolution genotyping of colorectal cancer in association with the detection of circulating tumor cells**. In., vol. 28, 2 edn: Springer Netherlands; 2011: 211-212.
40. Hoshino K, Huang Y-Y, Lane N, Huebschman M, Uhr JW, Frenkel EP, Zhang X: **Microchip-based immunomagnetic detection of circulating tumor cells**. *Lab on a chip* 2011, **11**(20):3449-3457.
41. Herold S, Gasiorowski L, Nowaczyk P, Schumann A, Theil G, Haubold K, Krahn T, Dyszkiewicz W, Murawa D, Lucke K: **An innovative approach for in-vivo isolation of circulating tumor cells (CTCs)**. In., vol. 49: Elsevier Ltd; 2013: S199.
42. Heitzer E, Auer M, Gasch C, Pichler M, Ulz P, Hoffmann EM, Lax S, Waldispuehl-Geigl J, Mauermann O, Lackner C *et al*: **Complex tumor genomes inferred from single circulating tumor cells by array-CGH and next-generation sequencing**. In., vol. 73, 10 edn. United States: American Association for Cancer Research Inc. (615 Chestnut Street, 17th Floor, Philadelphia PA 19106-3483, United States); 2013: 2965-2975.
43. Hayes DF, Smerage JB: **Circulating tumor cells**. In: *Progress in molecular biology and translational science.* vol. 95, C edn. United States: Academic Press Inc.; 2010: 95-112.
44. Hayes DF: **Blood biomarkers: A clinical point of view**. In., vol. 22: Oxford University Press; 2011: ii27.
45. Harris JL, Stocum M, Roberts L, Jiang C, Lin J, Sprott K: **Quest for the ideal cancer biomarker: An update on progress in capture and characterization of circulating tumor cells**. In., vol. 74, 2 edn. United States: Wiley-Liss Inc. (111 River Street, Hoboken NJ 07030-5774, United States); 2013: 138-147.
46. Harouaka RA, Zhou MD, Yeh YT, Khan WJ, Das A, Liu X, Christ CC, Dicker DT, Baney TS, Kaifi JT *et al*: **Flexible micro spring array device for**

high-throughput enrichment of viable circulating tumor cells. In., vol. 60, 2 edn. United States: American Association for Clinical Chemistry Inc. (2101 L Street NW, Suite 202, Washington DC 20037-1526, United States); 2014: 323-333.

47. Harb W, Fan A, Tran T, Danila DC, Keys D, Schwartz M, Ionescu-Zanetti C: **Mutational analysis of circulating tumor cells using a novel microfluidic collection device and qPCR assay**. In., vol. 6, 5 edn. United States: Translational Oncology Editorial Office (109 Zina Pitcher Place, BSRB - A628, Ann Arbor MI 48109-2200, United States); 2013: 528-538.

48. Guo W, Yang X-R, Sun Y-F, Shen M-N, Ma X-L, Wu J, Zhang C-Y, Zhou Y, Xu Y, Hu B *et al*: **Clinical Significance of EpCAM mRNA-Positive Circulating Tumor Cells in Hepatocellular Carcinoma by an Optimized Negative Enrichment and qRT-PCR-Based Platform**. *Clinical Cancer Research* 2014, **20**(18):4794-4805.

49. Grisanti S, Almici C, Consoli F, Buglione M, Verardi R, Bolzoni-Villaret A, Bianchetti A, Ciccarese C, Mangoni M, Ferrari L *et al*: **Circulating Tumor Cells in Patients with Recurrent or Metastatic Head and Neck Carcinoma: Prognostic and Predictive Significance**. *PloS one* 2014, **9**(8).

50. Gorges TM, Pantel K: **Circulating tumor cells as therapy-related biomarkers in cancer patients**. In., vol. 62, 5 edn. Germany: Springer Science and Business Media Deutschland GmbH (Heidelberger Platz 3, Berlin 14197, Germany); 2013: 931-939.

51. Gazzaniga P, Raimondi C, Gradilone A, Naso G, Cortesi E, Frati L: **Circulating tumor cells: Highlight on practical implications**. In., vol. 16, 1 edn. New Zealand: Adis International Ltd (41 Centorian Drive, Private Bag 65901, Mairangi Bay, Auckland 10 1311, New Zealand); 2012: 7-11.

52. Gazzaniga P, Naso G, Gradilone A, Cortesi E, Gandini O, Gianni W, Fabbri MA, Vincenzi B, di Silverio F, Frati L *et al*: **Chemosensitivity profile assay of circulating cancer cells: prognostic and predictive value in epithelial tumors**. *International Journal Of Cancer* 2010, **126**(10):2437-2447.

53. Gates JD, Benavides LC, Stojadinovic A, Mittendorf EA, Holmes JP, Carmichael MG, McCall S, Milford AL, Merrill GA, Ponniah S *et al*: **Monitoring circulating tumor cells in cancer vaccine trials**. In., vol. 4, 5 edn. United States: Landes Bioscience; 2008: 389-392.

54. Garcia-Paredes B, Segura E, Maestro M, Vidaurreta M, Sastre J, Diaz-Rubio E: **Circulating tumor cells: Clinical applicability**. In., vol. 6, 1 edn. Spain: Publicaciones Permanyer (Mallorca 310, Barcelona E-08037, Spain); 2011: 3-11.

55. Gao Y, Yuan Z: **Nanotechnology for the detection and kill of circulating tumor cells**. *Nanoscale Research Letters* 2014, **9**.

56. Gallant JN, Cheng H, Harouaka R, Zheng S, El-Deiry WS: **In vivo drug sensitivity testing of CTCs isolated using the FMSA device**. In., vol. 72, 8 SUPPL. 1 edn: American Association for Cancer Research Inc.; 2012.

57. Friedlander TW, Premasekharan G, Paris PL: **Looking back, to the future of**

circulating tumor cells. *Pharmacology & therapeutics* 2014, **142**(3):271-280.
58. Farace F: **Circulating tumor cells: Biologic challenges and clinical relevance**. In. Edited by Vielh P, Solomon D, Cochand-Priollet B, Schmitt F, vol. 57: S. Karger AG; 2013: 17.
59. Das A, Whitcomb TL, Allen JE, Dicker DT, Peters KL, El-Deiry WS: **Isolation and characterization of circulating tumor cells in a mouse model of colorectal cancer**. In., vol. 72, 8 SUPPL. 1 edn: American Association for Cancer Research Inc.; 2012.
60. Das A, Allen JE, Dicker DT, Peters KL, Joudeh J, El-Deiry WS: **Quantum dot multiplexing of prognostic marker and stem cell marker expression in colorectal cancer circulating tumor cells**. In., vol. 71, 8 SUPPL. 1 edn: American Association for Cancer Research Inc.; 2011.
61. Cummings J, Morris K, Zhou C, Sloane R, Lancashire M, Morris D, Bramley S, Krebs M, Khoja L, Dive C: **Method validation of circulating tumour cell enumeration at low cell counts**. *BMC cancer* 2013, **13**.
62. Coen JJ, Feldman AS, Smith MR, Zietman AL: **Watchful waiting for localized prostate cancer in the PSA era: what have been the triggers for intervention?** *Bju International* 2011, **107**(10):1582-1586.
63. Chan F, Goodman O, Fink L, Vogelzang NJ, Pomerantz D, Khoury JD: **Dramatically elevated circulating tumor cell numbers in a patient with small cell neuroendocrine carcinoma of the prostate**. *Archives of pathology & laboratory medicine* 2010, **134**(1):120-123.
64. Chan CML, Au TCC, Chan ATC, Ma BBY, Tsui NBY, Ng SSM, Hui EP, Chan LWC, Ho WS, Yung BYM *et al*: **Advanced technologies for studying circulating tumor cells at the protein level**. In., vol. 10, 6 edn. United Kingdom: Expert Reviews Ltd. (2 Albert Place, London N3 1QB, United Kingdom); 2013: 579-589.
65. Campion M, Zieman A, Voss J, Zimmerman C, Zhang J, Henry M, Halling K, Clayton A: **Correlation of circulating tumor cell enumeration between cytotechnologists and pathologists using the cellsearch1 assay**. In., vol. 118: John Wiley and Sons Inc.; 2010: 384-385.
66. Campion M, Zieman A, Voss J, Zimmerman C, Zhang J, Henry M, Halling K, Clayton A: **Correlation of Circulating Tumor Cell Enumeration between Cytotechnologists and Pathologists Using the CellSearch (R) Assay**. *Cancer Cytopathology* 2010, **118**(5):384-385.
67. Behbakht K, Sill MW, Darcy KM, Rubin SC, Mannel RS, Waggoner S, Schilder RJ, Cai KQ, Godwin AK, Alpaugh RK: **Phase II trial of the mTOR inhibitor, temsirolimus and evaluation of circulating tumor cells and tumor biomarkers in persistent and recurrent epithelial ovarian and primary peritoneal malignancies: A Gynecologic Oncology Group study**. *Gynecologic oncology* 2011, **123**(1):19-26.
68. Becker TM, Caixeiro NJ, Lim SH, Tognela A, Kienzle N, Scott KF, Spring KJ, de Souza P: **New frontiers in circulating tumor cell analysis: A reference guide for biomolecular profiling toward translational clinical use**.

International Journal Of Cancer 2014, **134**(11):2523-2533.
69. Armakolas A, Panteleakou Z, Nezos A, Tsouma A, Skondra M, Lembessis P, Pissimissis N, Koutsilieris M: **Detection of the circulating tumor cells in cancer patients**. In., vol. 6, 12 edn. United Kingdom: Future Medicine Ltd. (2nd Albert Place, Finchley Central, London N3 1QB, United Kingdom); 2010: 1849-1856.
70. Allard WJ, Matera J, Miller MC, Repollet M, Connelly MC, Rao C, Tibbe AGJ, Uhr JW, Terstappen LWMM: **Tumor cells circulate in the peripheral blood of all major carcinomas but not in healthy subjects or patients with nonmalignant diseases**. In., vol. 10, 20 edn. United States: American Association for Cancer Research Inc.; 2004: 6897-6904.
71. Alix-Panabieres C: **Detection and characterization of viable circulating tumor cells**. In., vol. 6, 11 SUPPL. 1 edn: American Association for Cancer Research Inc.; 2013.

3. Excluded Studies by full-text

(1): 51 not relevant or no sufficient data

1. Welinder C, Jansson B, Lindell G, Wenner J: **Cytokeratin 20 improves the detection of circulating tumor cells in patients with colorectal cancer**. *Cancer letters* 2014.
2. Steinert G, Scholch S, Niemietz T, Iwata N, Garcia SA, Behrens B, Voigt A, Kloor M, Benner A, Bork U *et al*: **Immune escape and survival mechanisms in circulating tumor cells of colorectal cancer**. In., vol. 74, 6 edn. United States: American Association for Cancer Research Inc.; 2014: 1694-1704.
3. Santos MF, Mannam VKR, Craft BS, Puneky LV, Sheehan NT, Lewis RE, Cruse JM: **Comparative analysis of innate immune system function in metastatic breast, colorectal, and prostate cancer patients with circulating tumor cells**. In., vol. 96, 3 edn. United States: Academic Press Inc.; 2014: 367-374.
4. Raimondi C, Nicolazzo C, Gradilone A, Giannini G, De Falco E, Chimenti I, Varriale E, Hauch S, Plappert L, Cortesi E *et al*: **Circulating tumor cells: Exploring intratumor heterogeneity of colorectal cancer**. In., vol. 15, 5 edn. United States: Landes Bioscience; 2014: 496-503.
5. Magni E, Botteri E, Ravenda PS, Cassatella MC, Bertani E, Chiappa A, Luca F, Zorzino L, Bianchi PP, Adamoli L *et al*: **Detection of circulating tumor cells in patients with locally advanced rectal cancer undergoing neoadjuvant therapy followed by curative surgery**. In., vol. 29, 9 edn. Germany: Springer Verlag; 2014: 1053-1059.
6. Kalikaki A, Politaki H, Souglakos J, Apostolaki S, Papadimitraki E, Georgoulia N, Tzardi M, Mavroudis D, Georgoulias V, Voutsina A: **KRAS**

genotypic changes of circulating tumor cells during treatment of patients with metastatic colorectal cancer. In., vol. 9, 8 edn; 2014.

7. Allen JE, Saroya BS, Kunkel M, Dicker DT, Das A, Peters KL, Joudeh J, Zhu J, El-Deiry WS: **Apoptotic circulating tumor cells (CTCs) in the peripheral blood of metastatic colorectal cancer patients are associated with liver metastasis but not CTCs**. In., vol. 5, 7 edn. United States: Impact Journals LLC; 2014: 1753-1760.
8. Neki K, Kawahara H, Watanabe K, Toyama Y, Akiba T, Yanaga K: **Usefulness of circulating tumor cells after preliminary chemotherapy for prediction of response to further anticancer therapy in patients with initially unresectable metastatic colorectal cancer**. In., vol. 33, 4 edn. Greece: International Institute of Anticancer Research (1st km Kapandritiou - Kalamou Rd., P.O. Box 22, Kapandriti, Attica GR-19014, Greece); 2013: 1769-1772.
9. Mostert B, Jiang Y, Sieuwerts AM, Wang H, Bolt-De Vries J, Biermann K, Kraan J, Lalmahomed Z, Van Galen A, De Weerd V *et al*: **KRAS and BRAF mutation status in circulating colorectal tumor cells and their correlation with primary and metastatic tumor tissue**. In., vol. 133, 1 edn. United States: Wiley-Liss Inc. (111 River Street, Hoboken NJ 07030-5774, United States); 2013: 130-141.
10. Kaifi JT, Kunkel M, Zhu J, Dicker DT, Gusani N, Yang Z, Sarwani NE, Li G, Kimchi ET, Staveley-O'Carroll KF *et al*: **Circulating tumor cells are associated with diffuse spread in stage IV colorectal cancer patients**. *Cancer biology & therapy* 2013, **14**(12):1174-1181.
11. Gasch C, Bauernhofer T, Pichler M, Langer-Freitag S, Reeh M, Seifert AM, Mauermann O, Izbicki JR, Pantel K, Riethdorf S: **Heterogeneity of epidermal growth factor receptor status and mutations of KRAS/PIK3CA in circulating tumor cells of patients with colorectal cancer**. In., vol. 59, 1 edn. United States: American Association for Clinical Chemistry Inc. (2101 L Street NW, Suite 202, Washington DC 20037-1526, United States); 2013: 252-260.
12. Ligthart ST, Coumans FAW, Bidard FC, Simkens LHJ, Punt CJA, de Groot MR, Attard G, de Bono JS, Pierga JY, Terstappen LWMM: **Circulating Tumor Cells Count and Morphological Features in Breast, Colorectal and Prostate Cancer**. In., vol. 8, 6 edn. United States: Public Library of Science (185 Berry Street, Suite 1300, San Francisco CA 94107, United States); 2013.
13. Rahbari NN, Bork U, Kircher A, Nimitz T, Scholch S, Kahlert C, Schmidt T, Steinert G, Ulrich AB, Reissfelder C *et al*: **Compartmental differences of circulating tumor cells in colorectal cancer**. In., vol. 19, 7 edn. United States: Springer New York (233 Spring Street, New York NY 10013-1578, United States); 2012: 2195-2202.
14. Leis O, Lopez-Mato P, Martin AG: **Circulating tumor stem cells as biomarkers for cancer progression**. In., vol. 2, 1 edn. Netherlands: Bentham Science Publishers B.V. (P.O. Box 294, Bussum 1400 AG, Netherlands); 2012: 36-53.

15. Gervasoni A, Sandri MT, Nascimbeni R, Zorzino L, Cassatella MC, Baglioni L, Panigara S, Gervasi M, Di Lorenzo D, Parolini O: **Comparison of three distinct methods for the detection of circulating tumor cells in colorectal cancer patients**. In., vol. 25, 6 edn. Greece: Spandidos Publications Ltd. (10 Vriaxidos Street, Athens 11635, Greece); 2011: 1669-1676.
16. Matsusaka S, Suenaga M, Mishima Y, Takagi K, Terui Y, Mizunuma N, Hatake K: **Circulating endothelial cells predict for response to bevacizumab-based chemotherapy in metastatic colorectal cancer**. *Cancer chemotherapy and pharmacology* 2011, **68**(3):763-768.
17. Thorsteinsson M, Soletormos G, Jess P: **Low number of detectable circulating tumor cells in non-metastatic colon cancer**. In., vol. 31, 2 edn. Greece: International Institute of Anticancer Research (1st km Kapandritiou - Kalamou Rd., P.O. Box 22, Kapandriti, Attiki GR-19014, Greece); 2011: 613-617.
18. Joosse S, Alix-Panabieres C, Deneve E, Beneken C, Nieuwland M, Kerkhoven R, Ramos J, Wikman H, Riethdorf L, Riethdorf S *et al*: **Genomic profiling of colorectal cancers from patients diagnosed with circulating tumor cells**. In., vol. 70, 8 SUPPL. 1 edn: American Association for Cancer Research Inc.; 2010.
19. Yalcin S, Kilickap S, Portakal O, Arslan C, Hascelik G, Kutluk T: **Determination of circulating tumor cells for detection of colorectal cancer progression or recurrence**. In., vol. 57, 104 edn. Greece: H.G.E. Update Medical Publishing Ltd. (P.O. Box 17257, Athens GR-10024, Greece); 2010: 1395-1398.
20. Wind J, Tuynman JB, Tibbe AG, Swennenhuis JF, Richel DJ, van Berge Henegouwen MI, Bemelman WA: **Circulating tumour cells during laparoscopic and open surgery for primary colonic cancer in portal and peripheral blood**. *European journal of surgical oncology : the journal of the European Society of Surgical Oncology and the British Association of Surgical Oncology* 2009, **35**(9):942-950.
21. Sastre J, Maestro ML, Puente J, Veganzones S, Alfonso R, Rafael S, Garcia-Saenz JA, Vidaurreta M, Martin M, Arroyo M *et al*: **Circulating tumor cells in colorectal cancer: Correlation with clinical and pathological variables**. In., vol. 19, 5 edn. United Kingdom: Oxford University Press; 2008: 935-938.
22. Balic M, Dandachi N, Hofmann G, Samonigg H, Loibner H, Obwaller A, van der Kooi A, Tibbe AGJ, Doyle GV, Terstappen L *et al*: **Comparison of two methods for enumerating circulating tumor cells in carcinoma patients**. *Cytometry Part B-Clinical Cytometry* 2005, **68B**(1):25-30.
23. Nikipelova EA, Kit OI, Novikova IA, Maksimov AY, Bakhtin AV, Nistratova OV, Ulianova EP, Anapalian V: **Probability of detection of circulating tumor cells in colorectal cancer stage III-IV**. In., vol. 32, 15 SUPPL. 1 edn: American Society of Clinical Oncology; 2014.
24. Zampino MG, Magni E, Ravenda SP, Botteri E, Bertani E, Chiappa A, Valvo

M, Zorzino L, Adamoli L, Nole F *et al*: **Detection of circulating tumor cells (CTCs) in stage T3-4 and/or N positive rectal cancer (RC) patients undergoing neoadjuvant therapy followed by curative surgery**. In., vol. 31, 15 SUPPL. 1 edn: American Society of Clinical Oncology; 2013.

25. Teijeira L, Viudez A, Antelo ML, Zalba S, Hernandez I, Herrera J, Tarifa A, Zazpe C, Vera R: **Circulating tumor cells (CTCs) as a surrogate biomarker of DFS in patients (pts) with liver-only (L) metastases of colorectal cancer (LM-CRC)**. In., vol. 49: Elsevier Ltd; 2013: S497.
26. Das A, Dicker DT, Harouaka R, Zheng S, Warfel N, Staveley-O'Carroll KF, El-Deiry WS, Kaifi JT: **Perioperative detection of circulating tumor cells in patients undergoing colorectal cancer liver and lung metastasectomy : Comparing CellSearch and FMSA technologies and investigating the role of EMT**. In., vol. 73, 8 SUPPL. 1 edn: American Association for Cancer Research Inc.; 2013.
27. Vay C, Sproll C, Will D, Baldus SE, Rehders A, Schulte Am Esch J, Topp SA, Knoefel WT, Stoecklein NH: **EpCAM as a surrogate marker for systemic adjuvant therapy-EpCAM-positive disseminated tumor cells represent an infrequent subpopulation in cancers of the digestive tract**. In., vol. 397, 4 edn: Springer Verlag; 2012: 654-655.
28. Scholten L, Terstappen LWMM, Van Der Palen J, Mastboom W, Tibbe A, Vermes I, De Groot M: **Circulating tumor cells as a possible prognostic tool in newly diagnosed nonmetastatic colorectal cancer?** In., vol. 30, 4 SUPPL. 1 edn: American Society of Clinical Oncology; 2012.
29. Mostert B, Sieuwerts AM, Van Galen AM, Bolt-de Vries J, Kraan J, Lalmahomed Z, Verhoef C, Jzermans JNMI, De Weerd V, Van Der Spoel P *et al*: **Generation of mRNA and miRNA gene expression profiles in circulating tumor cells of metastatic colorectal cancer patients**. In., vol. 72, 8 SUPPL. 1 edn: American Association for Cancer Research Inc.; 2012.
30. Das A, Joudeh J, Peters KL, Allen JE, Zheng S, Dicker DT, Yang Z, El-Deiry WS: **A population of metastatic colorectal cancer (mCRC) patients with radiologically proven liver metastasis and EpCAM expressing primary tumors show low circulating tumor cell counts by Veridex Cellsearch**. In., vol. 72, 8 SUPPL. 1 edn: American Association for Cancer Research Inc.; 2012.
31. Zogopoulos G, Selvarajah S, Ash C, Schwenter F, Chan D, Gallinger S, Moulton C: **Circulating tumor cells in patients undergoing liver resection for colorectal cancer liver metastases**. In., vol. 13: John Wiley and Sons Inc; 2011: 76.
32. Teijeira L, Viudez A, Arrazubi V, Verdun J, Uriz MJ, Antelo ML, Tarifa A, Zazpe C, Vera R: **Evolution of circulating tumor cells (CTCs) during neoadjuvant treatment and radical surgery in patients (pts) with liver-only metastases of colorectal cancer (L-CRC)**. In., vol. 29, 15 SUPPL. 1 edn: American Society of Clinical Oncology; 2011.
33. Chiappa A, Bertani E, Contino G, Uccelli F, Spampatti S, Zampino MG,

Leonardi C, Andreoni B: **Is there a role of circulating tumor cells (CTCs) as an independent prognostic factor in locally advanced rectal cancer?** In., vol. 47: Elsevier Ltd; 2011: S35.

34. Bork U, Rahbari NN, Kircher A, Niemetz T, Scholch S, Kahlert C, Steinert G, Buchler MW, Koch M, Weitz J: **Compartimental differences of circulating tumor cells in colorectal cancer**. In., vol. 396, 6 edn: Springer Verlag; 2011: 898.
35. Schwenter F, Ash C, Selvarajah S, Wegrynowski P, Zogopoulos G, O'Brien C, Moulton CA, Gallinger S: **Isolation of circulating tumor cells from hepatic vein blood of patients undergoing liver surgery for colorectal cancer metastases**. In., vol. 12: John Wiley and Sons Inc; 2010: 14.
36. Sato N, Hayashi N, Imamura Y, Kinoshita K, Kurashige J, Saito S, Karashima R, Hirashima K, Iwagami S, Miyamoto Y *et al*: **Usefulness of Transcription-Reverse Transcription Concerted (TRC) method in detecting circulating tumor cells of patients with colorectal cancer**. In., vol. 70, 8 SUPPL. 1 edn: American Association for Cancer Research Inc.; 2010.
37. Magni E, Botteri E, Cassatella MC, Bertani E, Barsalini C, Chiappa A, Bianchi P, Petrella M, Sandri M, Zampino MG: **Circulating tumor cells (CTCS) in locally advanced rectal cancer (LARC) patients undergoing neoadjuvant treatment**. In., vol. 21: Oxford University Press; 2010: viii223.
38. Chiappa A, Bertani E, Zampino M, Sandri M, Barsalini C, Andreoni B: **Detection of circulating tumor cells (CTCs) in locally advanced rectal cancer. Preliminary results**. In., vol. 36, 11 edn: W.B. Saunders Ltd; 2010: 1102.
39. Arrazubi V, Uriz M, Antelo M, Herrera J, Tarifa A, Zazpe C, Gomez M, Illarramendi J, Lainez N, Vera R: **Circulating tumor cells: Prognostic factor for metastatic colorectal cancer**. In., vol. 21: Oxford University Press; 2010: vi80.
40. Arrazubi V, M.J Ur, Antelo ML, Herrera J, Zazpe C, Tarifa A, Gomez ML, Salgado E, Herna ndez B, Vera R: **Circulating tumor cells in colorectal cancer. A prospective study**. In., vol. 21: Oxford University Press; 2010: viii213.
41. Chiappa AC, Contino G, Bertani E, Bianchi PP, Zampino MG, Sandri MT, Magni E, Branchi V, Corbellini C, Andreoni B: **Detection of circulating tumour cells in locally advanced colorectal cancer: preliminary data**. *Ejc Supplements* 2009, **7**(2):146-146.
42. Alix-Panabieres C, Riethdorf S, Deneve E, Mercier G, Nocca D, Vendrell J-P, Pantel K: **Quantitative assessment of circulating tumor cells in the mesenteric and peripheral blood of patients with colorectal cancer**. *Proceedings of the American Association for Cancer Research Annual Meeting* 2009, **50**:1208-1209.
43. Koopman M, Tol J, Miller MC, Terstappen LW, Tissing H, Van Den Eertwegh AJ, Bos MM, Imholz AL, Punt C: **Circulating tumor cells (CTC) in advanced colorectal cancer (ACC) patients undergoing 1st line treatment**

with chemotherapy, bevacizumab and cetuximab as an important and early predictor of survival. In., vol. 19, S8 edn: Oxford University Press; 2008: viii166.

44. Kaifi JT, Kunkel M, Allen JE, Dicker DT, Das A, Gusani NJ, Reed MF, Kimchi ET, Staveley-O'Carroll KF, El-Deiry WS: **Presence of circulating tumor cells in patients with resectable colorectal cancer lung metastases is a potential indicator for extrathoracic disease**. *Journal of Surgical Research* 2013, **179**(2).
45. 3Bao H, Burke PA, Huang J, Chen X, Brohawn PZ, Yao Y, Lechleider RJ, Sikorski RS, Buzoianu M, Zhang J *et al*: **Circulating Tumor Cells: Application as a Biomarker for Molecular Characterization and Predictor of Survival in an All-Comer Solid Tumor Phase I Clinical Study**. *PloS one* 2013, **8**(8).
46. Zhou L, Yang Z, Baker M, Dicker DT, El-Deiry WS: **Multispectral imaging of mismatch repair proteins in colorectal cancers and tumor cells isolated from blood**. *Cancer research* 2012, **72**(8).
47. Das A, Whitcomb TL, Allen JE, Dicker DT, Peters KL, El-Deiry WS: **Isolation and characterization of circulating tumor cells in a mouse model of colorectal cancer**. *Cancer research* 2012, **72**(8).
48. Coumans FAW, Ligthart ST, Terstappen LWMM: **All patients with metastatic breast, colorectal and prostate carcinoma have circulating tumor cells**. *Cancer research* 2012, **72**(8).
49. Soria J, De Braud FG, Cereda R, Bahleda R, Delmonte A, Angevin E, Varga A, Noberasco C, Dall'O E, Lassau N *et al*: **First-in-man study of E-3810, a novel VEGFR and FGFR inhibitor, in patients with advanced solid tumors**. *Journal of Clinical Oncology* 2011, **29**(15).
50. Das A, Allen JE, Dicker DT, Peters KL, Joudeh J, El-Deiry WS: **Quantum dot multiplexing of prognostic marker and stem cell marker expression in colorectal cancer circulating tumor cells**. *Cancer research* 2011, **71**(8).
51. Sua Villegas LF, Silva Perez NM, Vidaurreta Lazaro M, Maestro de las Casas ML, Rafael Fernandez S, Veganzones de Castro S: **Current and future techniques for quantifying circulating tumor cells: Importance in solid epithelial tumors**. *Revista del Laboratorio Clinico* 2011, **4**(3):163-169.

(2): 5 duplicated studies

1. Sastre J, Vidaurreta M, Gomez A, Rivera F, Massuti B, Lopez MR, Abad A, Gallen M, Benavides M, Aranda E *et al*: **Prognostic value of the combination of circulating tumor cells plus kras in patients with metastatic colorectal cancer treated with chemotherapy plus bevacizumab**. In., vol. 12, 4 edn. United States: Cancer Information Group, LP (3500 Maple Avenue, Suite 750, Dallas TX 75219, United States); 2013: 280-286.
2. Iwatsuki M, Toyoshima K, Watanabe M, Hayashi N, Ishimoto T, Eto K,

Iwagami S, Baba Y, Yoshida N, Hayashi A *et al*: **Frequency of HER2 expression of circulating tumour cells in patients with metastatic or recurrent gastrointestinal cancer**. *British journal of cancer* 2013, **109**(11):2829-2832.

3. Cohen SJ, Punt CJ, Iannotti N, Saidman BH, Sabbath KD, Gabrail NY, Picus J, Morse MA, Mitchell E, Miller MC *et al*: **Prognostic significance of circulating tumor cells in patients with metastatic colorectal cancer**. *Annals of oncology : official journal of the European Society for Medical Oncology / ESMO* 2009, **20**(7):1223-1229.
4. Cohen SJ, Punt CJ, Iannotti N, Saidman BH, Sabbath KD, Gabrail NY, Picus J, Morse M, Mitchell E, Miller MC *et al*: **Relationship of circulating tumor cells to tumor response, progression-free survival, and overall survival in patients with metastatic colorectal cancer**. *Journal of clinical oncology : official journal of the American Society of Clinical Oncology* 2008, **26**(19):3213-3221.
5. Matsusaka S, Suenaga M, Mishima Y, Kuniyoshi R, Takagi K, Terui Y, Mizunuma N, Hatake K: **Circulating tumor cells as a surrogate marker for determining response to chemotherapy in Japanese patients with metastatic colorectal cancer**. *Cancer science* 2011, **102**(6):1188-1192.

(3): 8 review or letter

1. Gazzaniga P, Gianni W, Raimondi C, Gradilone A, Lo Russo G, Longo F, Gandini O, Tomao S, Frati L: **Circulating tumor cells in high-risk nonmetastatic colorectal cancer**. In., vol. 34, 5 edn. Netherlands: Springer Netherlands (Van Godewijckstraat 30, Dordrecht 3311 GZ, Netherlands); 2013: 2507-2509.
2. Vegas H, Andre T, Bidard FC, Ferrand FR, Huguet F, Mariani P, Pierga JY: **Disseminated and circulating tumor cells in gastrointestinal oncology**. In., vol. 99, 5 edn. France: John Libbey Eurotext (127, avenue de la Republique, Montrouge 92120, France); 2012: 535-544.
3. Balic M, Lin H, Williams A, Datar RH, Cote RJ: **Progress in circulating tumor cell capture and analysis: Implications for cancer management**. In., vol. 12, 3 edn. United Kingdom: Expert Reviews Ltd. (2 Albert Place, London N3 1QB, United Kingdom); 2012: 303-312.
4. Riethdorf S: **Clinical role of detecting circulating tumor cells**. In., vol. 81, SUPPL1 edn. Ireland: Elsevier Ireland Ltd (P.O. Box 85, Limerick, Ireland); 2012: S132.
5. Gazzaniga P, Gianni W, Gradilone A, Raimondi C, Cortesi E, Frati L: **Circulating tumor cells in young and elderly patients with colorectal cancer: "Lupus et agnus"**. In., vol. 24, 6 edn. Italy: Springer International Publishing AG (Gewerbestrasse 11, Cham (ZG) 6330, Switzerland); 2012: 722-723.

6. Garcia-Paredes B, Maestro M, Vidaurreta M, Sastre J, Diaz-Rubio E: **Circulating tumor celles in colorectal cancer**. In., vol. 25, 3 edn. Spain: ARAN Ediciones S.A. (Castello 128 - 1o, Madrid 28006, Spain); 2011: 142-148.
7. Miller MC, Doyle GV, Terstappen LW: **Significance of Circulating Tumor Cells Detected by the CellSearch System in Patients with Metastatic Breast Colorectal and Prostate Cancer**. *Journal of oncology* 2010, **2010**:617421.
8. Davis JW: **Circulating tumor cell assays for the prognosis of prostate and colon cancers**. In., vol. 3, 3 edn. United Kingdom: Informa Healthcare (69-77 Paul Street, London EC2A 4LQ, United Kingdom); 2009: 293-301.

4. Included Studies

11 studies included in meta-analysis

1. Sotelo MJ, Sastre J, Maestro ML, Veganzones S, Vieitez JM, Alonso V, Gravalos C, Escudero P, Vera R, Aranda E *et al*: **Role of circulating tumor cells as prognostic marker in resected stage III colorectal cancer**. *Annals of oncology : official journal of the European Society for Medical Oncology / ESMO* 2014.
2. Seeberg LT, Waage A, Brunborg C, Hugenschmidt H, Renolen A, Stav I, Bjornbeth BA, Brudvik KW, Borgen EF, Naume B *et al*: **Circulating Tumor Cells in Patients With Colorectal Liver Metastasis Predict Impaired Survival**. *Annals of surgery* 2014.
3. Gazzaniga P, Raimondi C, Gradilone A, Biondi Zoccai G, Nicolazzo C, Gandini O, Longo F, Tomao S, Lo Russo G, Seminara P *et al*: **Circulating tumor cells in metastatic colorectal cancer: do we need an alternative cutoff?** *Journal of cancer research and clinical oncology* 2013, **139**(8):1411-1416.
4. Aggarwal C, Meropol NJ, Punt CJ, Iannotti N, Saidman BH, Sabbath KD, Gabrail NY, Picus J, Morse MA, Mitchell E *et al*: **Relationship among circulating tumor cells, CEA and overall survival in patients with metastatic colorectal cancer**. *Annals of oncology : official journal of the European Society for Medical Oncology / ESMO* 2013, **24**(2):420-428.
5. Kuboki Y, Matsusaka S, Minowa S, Shibata H, Suenaga M, Shinozaki E, Mizunuma N, Ueno M, Yamaguchi T, Hatake K: **Circulating tumor cell (CTC) count and epithelial growth factor receptor expression on CTCs as biomarkers for cetuximab efficacy in advanced colorectal cancer**. *Anticancer research* 2013, **33**(9):3905-3910.

6. Deneve E, Riethdorf S, Ramos J, Nocca D, Coffy A, Daures JP, Maudelonde T, Fabre JM, Pantel K, Alix-Panabieres C: **Capture of viable circulating tumor cells in the liver of colorectal cancer patients**. *Clinical chemistry* 2013, **59**(9):1384-1392.
7. Sastre J, Maestro ML, Gomez-Espana A, Rivera F, Valladares M, Massuti B, Benavides M, Gallen M, Marcuello E, Abad A *et al*: **Circulating tumor cell count is a prognostic factor in metastatic colorectal cancer patients receiving first-line chemotherapy plus bevacizumab: a Spanish Cooperative Group for the Treatment of Digestive Tumors study**. *The oncologist* 2012, **17**(7):947-955.
8. Sato N, Hayashi N, Imamura Y, Tanaka Y, Kinoshita K, Kurashige J, Saito S, Karashima R, Hirashima K, Nagai Y *et al*: **Usefulness of transcription-reverse transcription concerted reaction method for detecting circulating tumor cells in patients with colorectal cancer**. *Annals of surgical oncology* 2012, **19**(6):2060-2065.
9. Papavasiliou P, Fisher T, Kuhn J, Nemunaitis J, Lamont J: **Circulating tumor cells in patients undergoing surgery for hepatic metastases from colorectal cancer**. *Proceedings* 2010, **23**(1):11-14.
10. Tol J, Koopman M, Miller MC, Tibbe A, Cats A, Creemers GJ, Vos AH, Nagtegaal ID, Terstappen LW, Punt CJ: **Circulating tumour cells early predict progression-free and overall survival in advanced colorectal cancer patients treated with chemotherapy and targeted agents**. *Annals of oncology : official journal of the European Society for Medical Oncology / ESMO* 2010, **21**(5):1006-1012.
11. Hiraiwa K, Takeuchi H, Hasegawa H, Saikawa Y, Suda K, Ando T, Kumagai K, Irino T, Yoshikawa T, Matsuda S *et al*: **Clinical significance of circulating tumor cells in blood from patients with gastrointestinal cancers**. *Annals of surgical oncology* 2008, **15**(11):3092-3100.
